# Sequence and structural evolution of the KsgA/Dim1 methyltransferase family

**DOI:** 10.1186/1756-0500-1-108

**Published:** 2008-10-29

**Authors:** Heather C O'Farrell, Zhili Xu, Gloria M Culver, Jason P Rife

**Affiliations:** 1 Department of Medicinal Chemistry and Institute for Structural Biology and Drug Discovery, Virginia Commonwealth University, Richmond, Virginia 23219, USA; 2 Department of Biology, University of Rochester, Rochester, New York 14627, USA

## Abstract

**Background:**

One of the 60 or so genes conserved in all domains of life is the *ksgA/dim1 *orthologous group. Enzymes from this family perform the same post-transcriptional nucleotide modification in ribosome biogenesis, irrespective of organism. Despite this common function, divergence has enabled some family members to adopt new and sometimes radically different functions. For example, in *S. cerevisiae *Dim1 performs two distinct functions in ribosome biogenesis, while human mtTFB is not only an rRNA methyltransferase in the mitochondria but also a mitochondrial transcription factor. Thus, these proteins offer an unprecedented opportunity to study evolutionary aspects of structure/function relationships, especially with respect to our recently published work on the binding mode of a KsgA family member to its 30S subunit substrate. Here we compare and contrast KsgA orthologs from bacteria, eukaryotes, and mitochondria as well as the paralogous ErmC enzyme.

**Results:**

By using structure and sequence comparisons in concert with a unified ribosome binding model, we have identified regions of the orthologs that are likely related to gains of function beyond the common methyltransferase function. There are core regions common to the entire enzyme class that are associated with ribosome binding, an event required in rRNA methylation activity, and regions that are conserved in subgroups that are presumably related to non-methyltransferase functions.

**Conclusion:**

The ancient protein KsgA/Dim1 has adapted to cellular roles beyond that of merely an rRNA methyltransferase. These results provide a structural foundation for analysis of multiple aspects of ribosome biogenesis and mitochondrial transcription.

## Findings

Ribosome biogenesis is a fundamental process in all cells, requiring the consumption of large quantities of cellular resources under the control of an extraordinary level of regulation. In comparing prokaryotic and eukaryotic ribosome biogenesis pathways, the conservation of the KsgA/Dim1 family is unique. The presence and function of this enzyme has been maintained in every evolutionary lineage, including eukaryotic organelles.

KsgA catalyzes the conversion of two adjacent adenosines in the small subunit rRNA (A1518 and A1519 of 16S rRNA, *E. coli *numbering) to N6,N6-dimethyladenosines [1]. The KsgA family carries out this core methyltransferase function in all domains of life, including organelles, but has also added new roles as cellular organization became more complex. In eukaryotes, the KsgA ortholog Dim1 is essential for proper processing of the pre-18S small subunit rRNA [2], and Dim1 knockout is lethal. Pfc1, which is the KsgA ortholog found in chloroplasts of *Arabidopsis thaliana*, is important for chloroplast formation under chilling conditions [3].

A distinct eukaryotic ortholog, mtTFB, is transported into the mitochondria where, in addition to methylating the small subunit rRNA, it has adopted the ribosomally unrelated function of serving as a mitochondrial transcription factor [4,5]. In some mitochondria, there are two separate mtTFB proteins, mtTFB1 and mtTFB2, which are proposed to have arisen from a gene duplication event [5]. There is evidence that mtTFB1 has retained stronger methyltransferase activity, while mtTFB2 is more active as a transcription factor [5,6]. The fungi have only a single mtTFB, suggesting either loss of one of the paralogs in this lineage, or that the duplication occurred later in evolution. In at least one case, *S. cerevisiae*, the single mtTFB protein (sc-mtTFB) serves as a transcription factor but has lost its methyltransferase activity entirely [7]. sc-mtTFB lacks significant sequence homology to any of the KsgA/Dim1 enzymes; yeast mtTFBs are generally poorly conserved and difficult to identify via sequence homology [8].

Another important offshoot of the KsgA lineage is the Erm family of methyltransferases, which confer antibiotic resistance by methylating A2058 of the 23S rRNA [9]. The present-day Erm family almost certainly resulted from one or more gene duplications of a KsgA gene and subsequent evolution to permit recognition of a distinct target base [10].

KsgA's remarkable degree of conservation, coupled with the adaptation of new cellular functions, give us a unique opportunity to look at structural/function evolution of a single protein lineage. Previous studies have established the structural similarity between some rRNA adenosine dimethyltransferases [11,12]. Based on recent work in our groups we can now make predictions about how these proteins interact with their respective ribosomal targets. We also examine structural variations that may be important to the varied functions of KsgA orthologs and paralogs.

## Results

**Figure 1:**
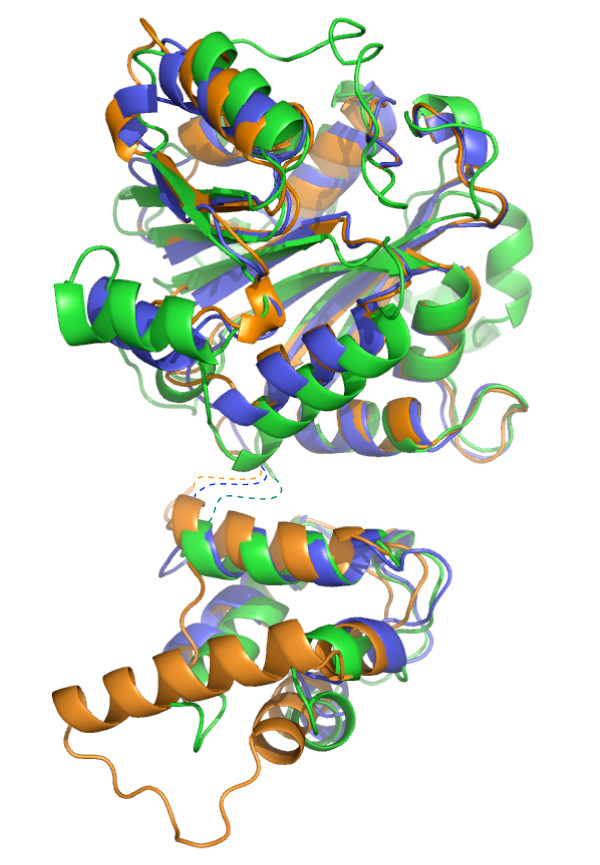
**Superimposition of KsgA, Dim1, and sc-mtTFB**. KsgA (PDB ID: 1QYR[12]) is shown in blue, Dim1 (PDB ID 1ZQ9; A. Dong, H. Wu, H. Zeng, P. Loppnau, M. Sundstrom, C. Arrowsmith, A. Edwards, A. Bochkarev and A. Plotnikov, unpublished data) in orange, and sc-mtTFB (PDB ID: 1I4W[11]) in green. These colors are retained in all figures. In each protein, the backbone was broken in the flexible linker between the N- and C-terminal domains (indicated by dashed lines); the two domains were separately aligned with KsgA using all residues in the domain. All figures were generated using the program Pymol [26].

*E. coli *KsgA [12], human Dim1 (hDim1; A. Dong, H. Wu, H. Zeng, P. Loppnau, M. Sundstrom, C. Arrowsmith, A. Edwards, A. Bochkarev and A. Plotnikov, unpublished data), *Plasmodium falciparum *Dim1 [13], and sc-mtTFB [11] have been characterized structurally. Figure 1 shows the structures of KsgA, hDim1 and sc-mtTFB superimposed. The three structures share the same overall architecture, with a few notable differences (discussed below).

Divergent orthologues of bacterial KsgA, including Dim1 from the eukaryote *S. cerevisiae *and the archaeon *Methanocaldococcus jannaschii *as well as h-mtTFB1 and h-mtTFB2, can all complement for KsgA function in *E. coli *[5,14,15]. Therefore, it is expected that these functional orthologues all interact with substrate ribosome particles in a similar manner. We recently reported a mode of binding for KsgA onto 30S subunits [16]. As was noted in this work, the binding site for KsgA on 30S involves protein interactions with 16S rRNA in helix 44 and in the 790 loop, but not in helix 45, the site of the methylated adenosines.

**Figure 2:**
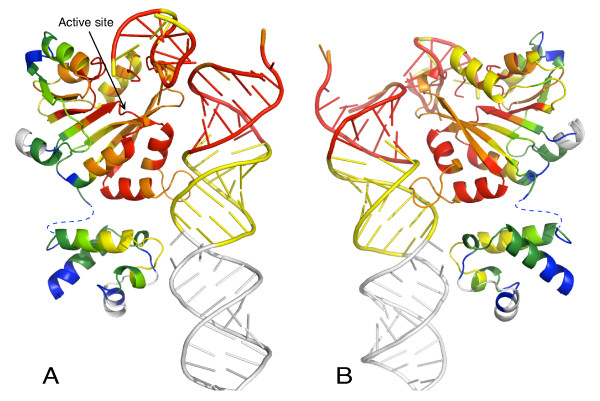
**Conservation in KsgA and helix 44 and the 790 loop**. The model of KsgA docked onto helix 44 and the 790 loop was taken from Xu *et al *[16]. RNA sequences that are conserved in greater than 98% of species are colored red, 90-90% in orange, and less than 90% in yellow; these sequences are present in at least 95% of organisms. White denotes sequences that are present in less than 95% of organisms. Residues in KsgA are colored by strength of alignment, with red denoting the most highly conserved regions, then orange, yellow, pale green, dark green and blue; white regions are the least conserved. Views shown in A and B are rotated approximately 180° about the vertical axis.

When 16S rRNA conservation is assessed across all three domains and the ribosome-containing organelles, regions that are important for translation show the highest evolutionary conservation [17]. Included in the set of highly conserved nucleotides are residues at the base of helix 44 and the 790 loop which interact with KsgA [16]. The high level of rRNA conservation at the KsgA binding site has likely constrained evolution of protein residues that interact with the rRNA, allowing evolutionarily divergent orthologues to recognize bacterial 30S subunits as a substrate. In order to assess KsgA conservation at the amino acid level, we aligned thirty-two KsgA, Dim1, mtTFB1 and Pfc1 proteins [see Additional file 1]. Since the mtTFB2 and mtTFB branches have become stronger transcription factors at the expense of the methyltransferase activity [5,6], we omitted these branches from this analysis. We used the program Expresso to construct the alignment and assess the conservation at each amino acid position [18], and then mapped the results onto the KsgA structure. RNA conservation was taken from the minimal ribosome modeled by Mears *et al *[17]. Figure 2 shows the patterns of conservation in KsgA and in helix 44 and the 790 loop. As expected, some of the most highly conserved residues in KsgA are found in the methyltransferase active site. Strikingly, there is also extensive conservation along the face of the protein that interacts with 16S rRNA, especially in the N-terminal domain. Conversely, the most poorly conserved residues are on the protein surface opposite its rRNA binding face.

The KsgA enzymes share the Rossman-like structural fold common to many SAM-dependent methyltransferases [12,19]. This fold consists of a seven-stranded beta sheet surrounded by a variable number of alpha helices, with well-defined binding pockets for SAM and the target nucleotide. In this way, the methyltransferase function has a clear structural basis. However, the structural basis for the other functions of the KsgA/Dim1 enzymes is unknown. Extensive sequence alignments of KsgA, Dim1 and mtTFB enzymes identify elements unique to each group. The addition of specific inserts relative to KsgA likely has permitted the eukaryotic Dim1 and mtTFB enzymes to gain functions while maintaining their roles as methyltransferases.

**Figure 3:**
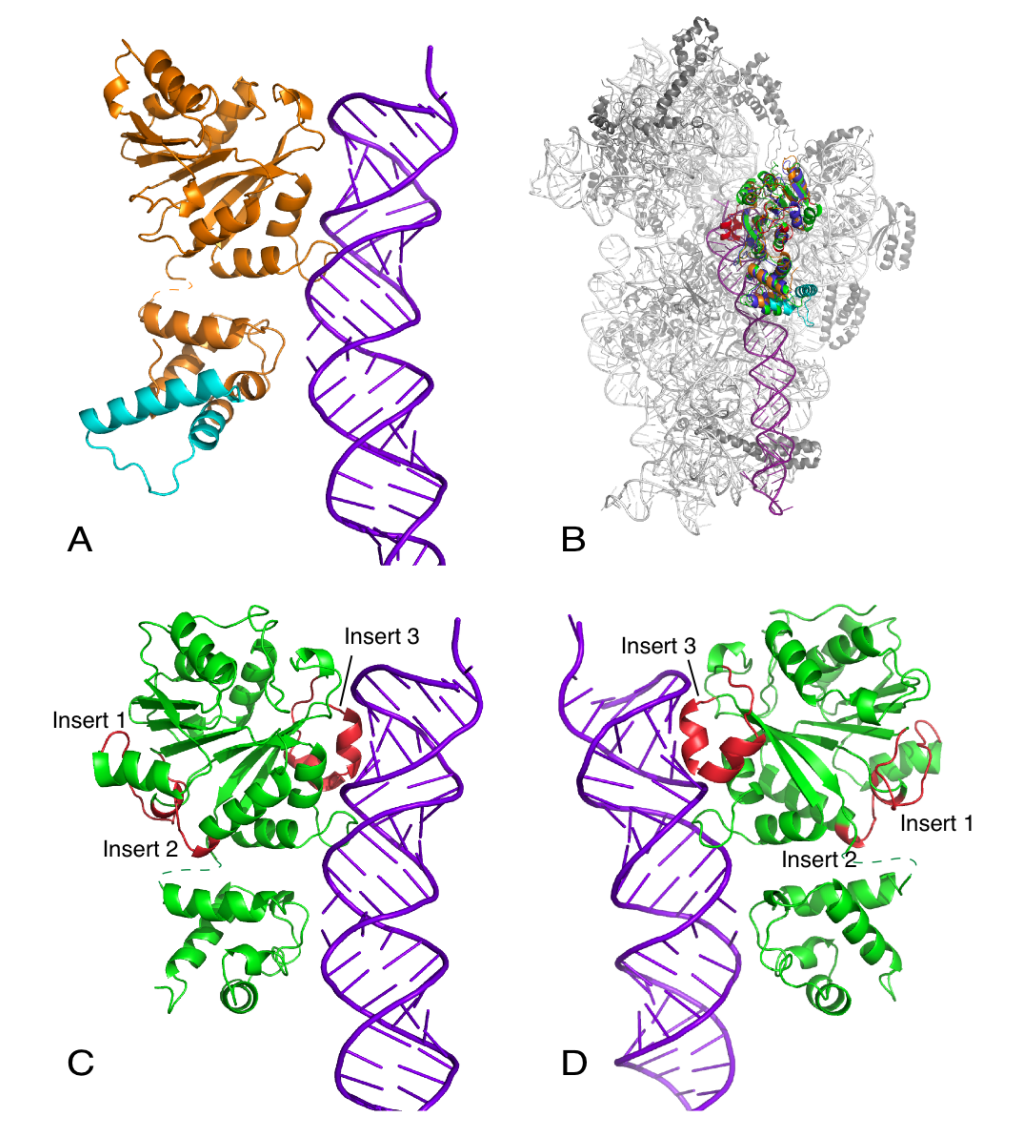
**Inserts in Dim1 and sc-mtTFB**. A. Dim1 modelled onto helix 44; the C-teminal insert is colored cyan. B. KsgA, Dim1 and sc-mtTFB modelled onto 30S subunits. C. and D. sc-mtTFB modelled onto helix 44; the N-terminal inserts are colored red and labelled. The two views represent approximately 180° rotation.

Eukaryotic Dim1 has a large insert in the C-terminal domain compared to KsgA (Figure 3a). Sequence within this region is only sparsely conserved, but there is a well-conserved region of 20–40 amino acids [see Additional file 1]. To visualize what impacts this insert might have on ribosome subunit binding, we superimposed the structure of h-Dim1 onto KsgA in our docked KsgA/30S subunit complex (Figure 3b). When Dim1 is bound to the small ribosomal subunit we predict that this insert makes no direct contact with the small ribosomal subunit. Therefore, the question naturally becomes what is its role in ribosome biogenesis. It is tempting to speculate that Dim1's essential function in rRNA processing is mediated by this "extra" region.

**Figure 4:**
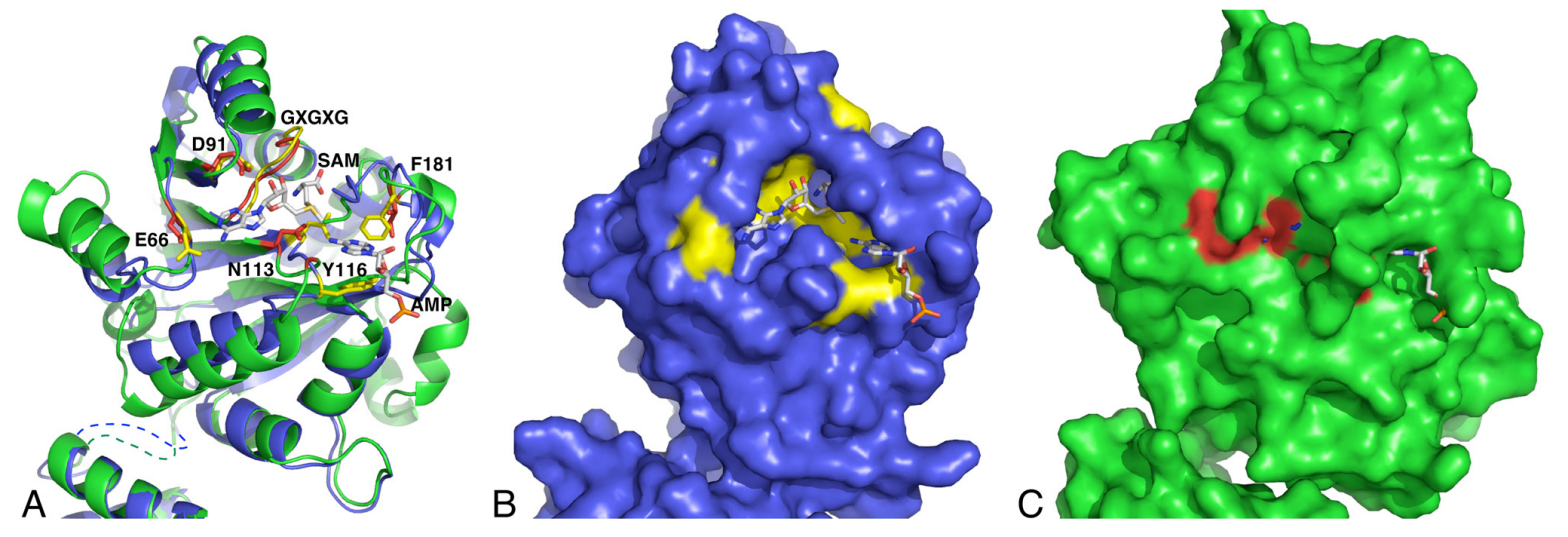
**The active sites of KsgA and sc-mtTFB**. A. Close up of the active sites. Residues in sc-mtTFB prior to residue 20 have been truncated, in order to start at a position analogous to the beginning of the KsgA crystal structure. KsgA is shown in blue, with residues mentioned in the text highlighted in yellow. sc-mtTFB is shown in green, with residues highlighted in red. SAM was modeled into the KsgA active site by using the co-crystal structure of ErmC' in complex with SAM (1QAO[23]) as a guide. Similarly, adenosine was modeled into the active site using the co-crystal structure of the DNA methyltransferase M. TaqI in complex with target DNA as a guide [27]. B. Surface representation of the KsgA active site. C. Surface representation of the sc-mtTFB active site.

A similar analysis can be performed by comparing KsgA and sc-mtTFB. mtTFB proteins have three inserts relative to KsgA proteins [20]; all of these inserts lie in the N-terminal domain (Figures 3c and 3d). We have superimposed sc-mtTFB onto the KsgA/30S model to evaluate what roles these inserts play, if any, in ribosome binding (Figure 3b). The first of these inserts is comprised of residues 116–130 of sc-mtTFB, and takes the form of a random coil leading into an extended beta strand; the second insert, residues 155–162, clusters together with insert 1 [20]. These two inserts, which are located on the opposite side of the protein from the 30S subunit, are generally conserved in all mtTFB proteins [see Additional file 2]; however, insert 1 is shorter in mtTFB1 than in mtTFB and mtTFB2, which have evolved as transcription factors perhaps at the expense of their full function as methyltransferases. Given that these two inserts do not appear as though they make direct interactions with the small ribosomal subunit, we suggest that they might be important to mtTFB's role as a transcription factor, perhaps comprising a single binding surface involved in transcription. The third insert, consisting of residues 209–230, is on the other side of the protein from the first two, and consists of two alpha-helices which form a long loop on the protein surface. Residues in this loop are important for binding of sc-mtTFB to the *S. cerevisiae *mitochondrial RNA polymerase, and this insert may also contribute to the protein's role as a transcription factor [21]. The presence of insert 3 is conserved in mtTFB and mtTFB2 proteins, but not in mtTFB1 proteins [see Additional file 2]. Notably, mtTFB insert 3 is in a position to interact with the 30S subunit, either in a positive or a negative manner.

sc-mtTFB, and possibly other fungal TFB proteins, have lost their methyltransferase activity during their evolution as transcription factors [7]. Figure 4 highlights the structural explanation for this loss of function. KsgA and Dim1 both have a well-defined active site pocket, with clear binding sites for the SAM methyl donor and the target adenosine. The active site, which is essentially the same in the Erm enzymes [22-24], is homologous to other adenosine methyltransferases, and has been well-conserved. However, the area of the active site pocket has diverged in sc-mtTFB [11]. sc-mtTFB retains residues important for SAM binding, including the canonical GXG motif, two acidic residues which stabilize SAM in the binding site, and an asparagine residue which interacts with SAM [11] (Figure 4a). However, the SAM binding pocket, which is clearly defined in KsgA (Figure 4b), is occluded in sc-mtTFB (Figure 4c), mainly by the side chains of residues Y22, Y54 and R79, which fill in the pocket. Similarly, the adenosine binding pocket in KsgA/Dim1 is not present in sc-mtTFB (Figure 4c), with that space being occupied by the side chains of residues K21, T139 and K240. Also, two aromatic residues that stabilize the target base, corresponding to residues Y116 and F181 in KsgA, are not found in the sc-mtTFB active site (Figure 4a).

**Figure 5:**
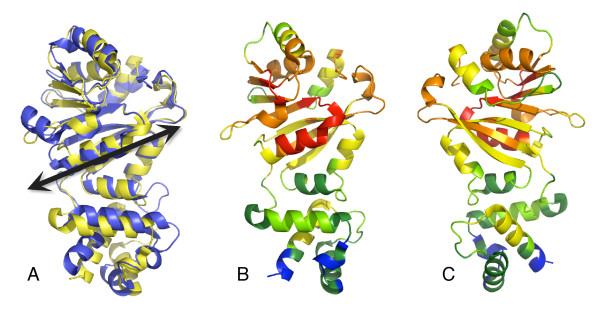
**Conservation in the Erm family**. A. Superimposition of ErmC' (PDB ID: 1QAM[23] and KsgA. ErmC' is colored yellow. B. and C. Conservation of protein residues mapped onto the ErmC' structure Residues are colored by strength of alignment, with red denoting the most highly conserved regions, then orange, yellow, pale green, dark green and blue; white regions are the least conserved. The two views represent approximately 180° rotation.

Structural comparison of KsgA and ErmC' shows that the two proteins are very similar, with the most notable divergence being in the C-terminal domains (Figure 5a). These two enzymes perform the same chemistry, dimethylation of adenosine, and share an active site structure common to enzymes that catalyze this reaction [12,23]. However, the Erm enzymes have evolved to recognize a distinct target, A2058 in 23S rRNA [9], and have thus been subject to different selective pressures in terms of substrate binding. While KsgA's RNA-binding surface extends along the length of the protein, ErmC' has been proposed to bind its target RNA across the cleft formed by the N- and C-terminal domains [25]. We constructed an alignment of 31 Erm sequences [see Additional file 3] and mapped conservation onto the structure of ErmC' from *B. subtilis *[23]; the results are shown in Figure 5(b) and 5(c). Like KsgA, the active site contains some of the most highly conserved residues. However, the only other major conserved area is a helix in the N-terminal domain, along the cleft that forms the proposed RNA binding site. The Erm family's binding mode allows these enzymes to methylate a small RNA hairpin [23]; this lies in sharp contrast to KsgA's complex substrate requirements, which involve binding to an RNA helix distal from the site of its enzymatic activity [16]. This dichotomy highlights the plasticity of the core KsgA structure, which has allowed orthologous and paralogous proteins to fill multiple and distinct roles with only modest adaptive changes.

## Conclusion

To our knowledge, the KsgA proteins are unique in both their level of conservation and their functional flexibility. KsgA was one of the first rRNA methyltransferases to be identified and has received episodic scrutiny over the last thirty years. Despite a collection of important observations, a thorough understanding of this protein family's role in ribosome biogenesis has been elusive. Certainly, the KsgA/Dim1 family has significance beyond the methyltransferase activity that was first described. Sequence and structural comparisons of these proteins, in concert with data about the binding of KsgA to the 30S subunit, implicate certain regions that might be important to the various functions of KsgA orthologs. Further experiments will help define individual characteristics of this important family of proteins.

## Competing interests

The authors declare that they have no competing interests.

## Authors' contributions

All authors discussed results and contributed to the manuscript. HCO compiled sequences and prepared alignments. JPR docked proteins onto the 30S subunit structure.

## Supplementary Material

Additional file 1 **Sequence alignment of KsgA orthologs**. Archaeal, eukaryotic, and mitochondrial KsgA orthologues were identified by performing a genomic BLAST search using the *E. coli *protein sequence (accession number P06992) as the query sequence. Organisms were chosen to represent a broad evolutionary diversity of species. The structure-based sequence alignment was perfomed using the program Expresso [18]. Structures used for the alignment were 1QYR[12], 1ZQ9 (A. Dong, H. Wu, H. Zeng, P. Loppnau, M. Sundstrom, C. Arrowsmith, A. Edwards, A. Bochkarev and A. Plotnikov, unpublished data), and 2H1R[13]. Organisms represented are as follows. Eukaryotes: *Arabidopsis thaliana *(at), *Dictyostelium discoideum *(dd), *Leishmania brazilensis *(lb), *Giardia lamblia *(gl), *Plasmodium vivax *(pv), *Homo sapiens *(hs), *Saccharomyces cerevisiae *(sc), *Drosophila melanogaster *(dm), and *Caenorhabditis elegans *(ce). Archaea: *Methanopyrus kandleri *(mk), *Methanosaeta thermophila *(mth), *Haloquadratum walsbyi *(hw), *Methanoculleus marisnigri *(mma), *Methanocaldococcus jannaschii *(mj), *Pyrococcus horikoshii *(ph), *Methanosphaera stadtmanae *(ms), *Picrophilus torridus *(pt), *Archaeoglobus fulgidus *(af), *Aeropyrum pernix *(ap), *Sulfolobus solfataricus *(ss), *Pyrobaculum aerophilum *(pa), and *Cenarchaeum symbiosum *(cs). Bacteria: *Synechococcus elongatus *(se), *Bacillus subtilis *(bs), *Mycobacterium tuberculosis *(mtu), *Thermus thermophilus *(tt), *Bacteroides fragilis *(bf), *Chlamydia trachomatis *(ct), *Borrelia burgdorferi *(bb), and *Escherichia coli *(ec). Accession numbers for each sequence are found in Additional file 4. Click here for file

Additional file 2 **Sequence alignment of mtTFB, mtTFB1, and mtTFB2 proteins**. The structure-based sequence alignment was perfomed using the program Expresso [18]. The structure 1I4W[11] was used for the alignment. Organisms represented are *Caenorhabditis elegans *(ce), *Homo sapiens *(hs), *Drosophila melanogaster *(dm), *Anopheles gambiae *(ag), *Apis mellifera *(am), *Xenopus laevis *(xl), *Takifugu rubripes *(tr), *Ciona intestinalis *(ci), *Rattus norvegicus *(rn), *Pan troglodytes *(pt), *Mus musculus *(mmu), *Bos taurus *(bt), *Tetraodon nigroviridis *(tn), *Saccharomyces cerevisiae *(sc), *Schizosaccharomyces pombe *(sp), *Kluyveromyces lactis *(kl), *Eremothecium gossypii *(eg), *Candida albicans *(ca), *Dictyostelium discoideum *(dd), *Trypanosoma brucei *(tb), and *Leishmania major *(lm). Accession numbers for each sequence are found in Additional file 4. Click here for file

Additional file 3 **Sequence alignment of Erm enzymes**. Erm enzymes were identified using the Nomenclature Center for MLS Genes, maintained by Dr. Marilyn C. Roberst [28]. One member of each class was chosen, with two exceptions. ErmI was not used because a corresponding sequence could not be found. Erm32 was not used because this enzyme methylates G748 rather than A2058 [29]. The structure based sequence alignment was performed with Expresso [18]. Structures used for the alignment were 1QAM[23] and 1YUB[24]. Organisms represented are *Staphylococcus aureus, Enterococcus faecalis, Bacillus subtilis, Bacillus licheniformis, Saccharopolyspora erythraea, Bacteroides fragilis, Lysinibacillus sphaericus, Streptomyces thermotolerans, Streptomyces fradiae, Streptomyces coelicolor, Clostridium perfringens, Aeromicrobium erythreum, Lactobacillus reuteri, Streptomyces lincolnensis, Streptomyces viridochromogenes, Micromonospora griseorubida, Corynebacterium jeikeium, Streptomyces ambofaciens, Streptomyces venezuelae, Staphylococcus sciuri, Bacillus clausii, Bacteroides coprosuis, Micrococcus luteus, Mycobacterium tuberculosis, Mycobacterium smegmatis, Mycobacterium fortuitum, Mycobacterium mageritense, and Mycobacterium abscessus*, Accession numbers are found in Additional file 4. Click here for file

Additional file 4 **Sequences used in protein alignments**. Sequences were compiled from NCBI and Ensembl; organisms and accession numbers are indicated. Click here for file
