# Supplementary material for: Sequence and structural evolution of the KsgA/Dim1 methyltransferase family
Source: BMC Res Notes. 2008 Oct 29;1:108. doi: 10.1186/1756-0500-1-108 (PMC2614427; doi:10.1186/1756-0500-1-108)
Supplement: Additional file 1 — Sequence alignment of KsgA orthologs. Archaeal, eukaryotic, and mitochondrial KsgA orthologues were identified by performing a genomic BLAST search using the E. coli protein sequence (accession number P06992) as the query sequence. Organisms were chosen to represent a broad evolutionary diversity of species. The structure-based sequence alignment was perfomed using the program Expresso [18]. Structures used for the alignment were 1QYR[12], 1ZQ9 (A. Dong, H. Wu, H. Zeng, P. Loppnau, M. Sundstrom, C. Arrowsmith, A. Edwards, A. Bochkarev and A. Plotnikov, unpublished data), and 2H1R[13]. Organisms represented are as follows. Eukaryotes: Arabidopsis thaliana (at), Dictyostelium discoideum (dd), Leishmania brazilensis (lb), Giardia lamblia (gl), Plasmodium vivax (pv), Homo sapiens (hs), Saccharomyces cerevisiae (sc), Drosophila melanogaster (dm), and Caenorhabditis elegans (ce). Archaea: Methanopyrus kandleri (mk), Methanosaeta thermophila (mth), Haloquadratum walsbyi (hw), Methanoculleus marisnigri (mma), Methanocaldococcus jannaschii (mj), Pyrococcus horikoshii (ph), Methanosphaera stadtmanae (ms), Picrophilus torridus (pt), Archaeoglobus fulgidus (af), Aeropyrum pernix (ap), Sulfolobus solfataricus (ss), Pyrobaculum aerophilum (pa), and Cenarchaeum symbiosum (cs). Bacteria: Synechococcus elongatus (se), Bacillus subtilis (bs), Mycobacterium tuberculosis (mtu), Thermus thermophilus (tt), Bacteroides fragilis (bf), Chlamydia trachomatis (ct), Borrelia burgdorferi (bb), and Escherichia coli (ec). Accession numbers for each sequence are found in Additional file 4. [file 1756-0500-1-108-S1.pdf]

T-COFFEE, Version_5.31Fri Oct 26 17:01:36 2007
Cedric Notredame
CPU TIME:119 sec.
SCORE=42
*
BAD AVG GOOD
*

atDim1	:	47
ddDim1	:	46
lbDim1	:	43
glDim1	:	44
pvDim1	:	44
hsDim1	:	47
scDim1	:	44
atPFC1	:	40
dmTFB1	:	39
ceTFB1	:	38
hsTFB1	:	39
mkDim1	:	44
mthDim1	:	45
hwDim1	:	42
mmaDim1	:	43
mjDim1	:	43
phDim1	:	43
msDim1	:	41
ptDim1	:	43
afDim1	:	46
apDim1	:	38
ssDim1	:	29
paDim1	:	43
csDim1	:	37
seKsgA	:	43
bKsgA	:	43
mtuKsgA	:	40
ttKsgA	:	41
bfKsgA	:	44
ctKsgA	:	40
bbKsgA	:	41
ecKsgA	:	44
cons	:	42

```
atDim1     1  M-----------------AGGKIRKEK------------------------  10
ddDim1     1  -------------------------------------------MVKPVKIG   8
lbDim1     1  -MPKEPRAVP-MGIICAEPIHAASRK------------------IKFSTKV  31
glDim1     1  ---------------------------------------------------   0
pvDim1     1  -MRAGGLALL-RNLQNALSSSSAAKMKPKRGYHQGVANKPVS-ILKNVSDA  48
hsDim1     1  -MPKV----------------------------------------------   4
scDim1     1  -MGKA----------------------------------------------   4
atPFC1     1  -------------MMNAVITSATINCNSLSPSWTCGDNSPSKLLLGEISAA  38
dmTFB1     1  MAQPSARVLQSGMRLP-----------------------------------  16
ceTFB1     1  ------MA--SASRLP-----------------------------------   8
hsTFB1     1  -MAASGKLS--TCRLP-----------------------------------  13
mkDim1     1  ---------------------------------------------------   0
mthDim1    1  ---------------------------------------------------   0
hwDim1     1  -MTDA------------TSG-----------------------SDPDSTTP  15
mmaDim1    1  ---------------------------------------------------   0
mjDim1     1  ---------------------------------------------------   0
phDim1     1  -MSSR------------------IRINTK----------------------  10
msDim1     1  ---------------------------------------------------   0
ptDim1     1  ---------------------------------------------------   0
afDim1     1  ---------------------------------------------------   0
apDim1     1  MPP--GSG--RGGRRR-----------------------------------  12
ssDim1     1  ---------------------------------------------------   0
paDim1     1  ---------------------------------------------------   0
csDim1     1  ---------------------------------------------------   0
seKsgA     1  ---------------------------------------------------   0
bKsgA      1  ----------MNKDIA-----------------------------------   6
mtuKsgA    1  MCCTSGCAL--TIRLL-----------------------------------  14
ttKsgA     1  -----------MSKLA-----------------------------------   5
bfKsgA     1  ---------------------------------------------------   0
ctKsgA     1  -MARS----------------------------------------------   4
bbKsgA     1  -MILSLLS--MNINYN-----------------------------------  13
ecKsgA     1  ---------------------------------------------------   0

cons       1                                                       51
```

```
atDim1     11 ----------------------PKASNRAPSNHYQGGISFHKSKGQHILKN  39
ddDim1      9 VDAKV-------------------EKTKSATAARHHEFQMNKSYGQHLLKN  40
lbDim1     32 ITLGKSTPQG-HRRGAAAAGRQEKAGPGVRAGGSQSGIVFNKGFGQHILKN  81
glDim1      1 ----------------------------------MQGFELTKQHGQHLLAN  17
pvDim1     49 IGKKKSAPQG-GNASRHISTSKPK-------SGNKMNMILYKKHGQHLLKN  91
hsDim1      5 ----KSGAIG-RRRGR---------QEQRRELKSAGGLMFNTGIGQHILKN  41
scDim1      5 ----AKKKYS-G---------ATSSKQVSAEKHLSSVFKFNTDLGQHILKN  41
atPFC1     39 LSRRRTVKVSCGKSSPDDYHSTL-------KSLNSRGRFPRKSLGQHYMLN  82
dmTFB1     17 ------------------------PMPTIRELVKLYRLQARKQLSQNFLMD  43
ceTFB1      9 ------------------------PLPALRDFIHMYRLRAKKILSQNYLMD  35
hsTFB1     14 ------------------------PLPTIREIIKLLRLQAAKQLSQNFLLD  40
mkDim1      1 ----------------------MRSVEYLAYLRSKYGIRPRRRLGQHFMVD  29
mthDim1     1 -----------------------------------------MKSGQHFLTD  10
hwDim1     16 VDLT------------------GEDFRDPDALRRRAGVSGDPNFDQHFLID  48
mmaDim1     1 ---------------------------------------MSAPRDQHFLVD  12
mjDim1      1 ------------------------------------MFKPKKKLGQCFLID  15
phDim1     11 ----SLLVQP-GYSGS-------KMRDRLFFLLSKYGIRPRDSIGQHFLII  49
msDim1      1 -------------------------MSNTKEILEKYNIKLDTNKSQNYLID  26
ptDim1      1 -------------------------------------MKFSRKYGQVFLKN  14
afDim1      1 -------------------------------------MKLRKSLGQHMLVD  14
apDim1     13 ------------------------AESLVREVLGLAGLRPSDRLGQHFLID  39
ssDim1      1 -------------------------------------------------MD   2
paDim1      1 --------------------------------------MKRRRLAQHFLRD  13
csDim1      1 ------------------------------------------------MRS   3
seKsgA      1 -------------------------------------MPARKRFGQHWLRS  14
bKsgA       7 ------------------------TPIRTKEILKKYGFSFKKSLGQNFLID  33
mtuKsgA    15 ------------------------GRTEIRRLAKELDFRPRKSLGQNFVHD  41
ttKsgA      6 ------------------------SPQSVRALLERHGLFADKRFGQNFLVS  32
bfKsgA      1 ----------------------------------MKLVKPKKFLGQHFLKD  17
ctKsgA      5 ------------------------SIEQLTSFLRSVNGRAKKALSQNFLVD  31
bbKsgA     14 ------------------------SITSIKQTLKERKIAPRKLWGQNYLIN  40
ecKsgA      1 -----------------------------MNNRVHQGHLARKRFGQNFLND  22

cons       52                                                    102
```

```
atDim1      40  PLLVDSIVQK---AGIKSTDVILEIGPGTGNLTKKLLEA-GKEVIAVELDS   86
ddDim1      41  PLIIDAIVDK---SQLKSTDTVLEIGPGTGNLTMKLLEN-CKKVIAIEVDP   87
lbDim1      82  PLVIAAIVEK---SAIKPTDVVVEIGPGTGNLTEKLLQT-AKKVIAFEIDP  128
glDim1      18  PLVIKSIVEK---AEIRSTDTVLEIGPGTGNLTLALLEK-ARHVIAIEIDP   64
pvDim1      92  PGILDKILLA---AKIKSSDVVLEIGCGTGNLTVKLLPI-AKKVITIDIDA  138
hsDim1      42  PLIINSIIDK---AALRPTDVVLEVGPGTGNMTVKLLEK-AKKVVACELDP   88
scDim1      42  PLVAQGIVDK---AQIRPSDVVLEVGPGTGNLTVRILEQ-AKNVVAVEMDP   88
atPFC1      83  SDINDQLASA---ADVKEGDFVLEIGPGTGSLTNVLINL-GATVLAIEKDP  129
dmTFB1      44  ERLTDKIVKSA--GRIDPRDLVLEVGPGPGGITRSILRRHPQRLLLVEKDP   92
ceTFB1      36  MNITRKIAKH---AKVIEKDWVIEIGPGPGGITRAILEAGASRLDVVEIDN   83
hsTFB1      41  LRLTDKIVRK---AGNLTNAYVYEVGPGPGGITRSILNADVAELLVVEKDT   88
mkDim1      30  DNILEFMVEA---AEVREDDIVLEIGPGPGLLTRYLMTR-AGQVIAVELDG   76
mthDim1     11  RGIAERIAGY---AEISPSDRILEIGPGKGSLTEFLAAR-AGRVYAIEADP   57
hwDim1      49  DRVLDRIPTYL--LDSTDTTHILEIGAGTGALTDRLLAV-GDTVTVIERDA   96
mmaDim1     13  RRAVEKIAGF---VDVS-GRRVLEIGPGEGILTRALLDR-DADVIAVEIDP   58
mjDim1      16  KNFVNKAVES---ANLTKDDVVLEIGLGKGILTEELAKN-AKKVYVIEIDK   62
phDim1      50  EDVIEKAIET---ANVNENDVILEVGPGLGFLTDELAKR-AKKVYTIEIDQ   96
msDim1      27  DNKLNIILEN---ADIQDNETILEIGAGIGTLTLPMAKK-AKKVIAIEKDP   73
ptDim1      15  LNIAKIEVNL---LNLSPGERVLEIGPGHGILTSIIMEK-NVNLTVVEPDH   61
afDim1      15  RRVISRIVGY---AELSEDDVVLEVGCGTGNLTSALLRK-C-SVVGIEKDP   60
apDim1      40  DRAVGEFLKPLEKAAAEGIREALEIGPGAGSITLPAAEV-LDRIVAVELDN   89
ssDim1       3  NGI-----------------RPILEIGCGKGNITRFL-----EPDICIELDD   32
paDim1      14  PSVAEYIA-----GLVPSGLDVIEVGPGAGALTIPLAKR-SKTVYAIEIDK   58
csDim1       4  RQAARRIADS---AGISPGDTVLEVGTGLGALTRELCGR-GARIISVERNG   50
seKsgA      15  EAILDRIVAA---AELRPSDRVLEIGPGRGALTQRLLAA-VDGLVAVELDR   61
bKsgA       34  TNILNRIVDH---AEVTEKTGVIEIGPGIGALTEQLAKR-AKKVVAFEIDQ   80
mtuKsgA     42  ANTVRRVVAA---SGVSRSDLVLEVGPGLGSLTLALLDR-GATVTAVEIDP   88
ttKsgA      33  EVHLRRIVEA---ARPF-TGPVFEVGPGLGALTRALLEA-GAEVTAIEKDL   78
bfKsgA      18  LKVAQDIADT---VDTFPDLPILEVGPGMGVLTQFLVKK-ERLVKVVEVDY   64
ctKsgA      32  GNILRKILTT---AEVQPGDWVLEIGPGFGALSEVLLSQ-GANVIALEKDP   78
bbKsgA      41  ESIRQKIIES---LDIKENEKIWEIGPGLGAMTEILLKK-TNLLTAFEIDL   87
ecKsgA      23  QFVIDSIVSA---INPQKGQAMVEIGPGLAALTEPVGER-LDQLTVIELDR   69

cons       103                         *:* * . ::             : :    153
```

```
atDim1     87  RMVLELQRRFQGTP--FSNRLKVIQGDVLKT----------------ELP-  118
ddDim1     88  RMAAELQKRVAASP--YAQHLQIILGDFLKV----------------DLP-  119
lbDim1    129  RMVAELNKRFQNTP--LASKLQIIRGNCLEQ----------------DFP-  160
glDim1     65  RMVSELKKRIAAIPE-YRGKFTIIHKDFTKMPPS-------------EIP-  100
pvDim1    139  RMVSEVKKRCLYEG--Y-NNLEVYEGDAIKT----------------VFP-  169
hsDim1     89  RLVAELHKRVQGTP--VASKLQVLVGDVLKT----------------DLP-  120
scDim1     89  RMAAELTKRVRGTP--VEKKLEIMLGDFMKT----------------ELP-  120
atPFC1    130  HMVDLVSERFAGS-----DKFKVLQEDFVKCHIRSHMLSILETRR-LSHPD  174
dmTFB1     93  RFGETLQLLKECASP-LNIQFDIHYDDILRFNIEQHIPDTSQ---------  133
ceTFB1     84  RFIPPLQHLAEAAD----SRMFIHHQDALRTEIGDIWKNETARPESVDWHD  130
hsTFB1     89  RFIPGLQMLSDAAP----GKLRIVHGDVLTFKVEKAFSESLKRPWE-DDP-  133
mkDim1     77  RMVEILKRELGEA-----PNLEIVRADFLEY----------------DVPD  106
mthDim1    58  ELARYVEESF--------PNVEVIQGDALRV----------------DLP-   83
hwDim1     97  TLAAFLREEFAVMI--DDGRLNIIEGDALEV----------------TLP-  128
mmaDim1    59  ALVEELEIAFADEI--GEGRLEIIRGDAKKV----------------DIP-   90
mjDim1     63  SLEPYANKLKE-----LYNNIEIIWGDALKV----------------DLNK   92
phDim1     97  KIIEILKKEYSW------NNVKIIQGDAVRV----------------EWP-  124
msDim1     74  IIVDILKQQIIKEK---LTNIEIIKDDALKV----------------DFP-  104
ptDim1     62  RFYNEIILRFPG--------LNAIKNSFLDL----------------NPG-   87
afDim1     61  LMVKRLRERFSDFI--GKGRFRLIQGDALKV----------------DFP-   92
apDim1     90  RLASALSRLA-------PARVAVITGDGVSH---------------AAAS-  117
ssDim1     33  KMIEYLKN------------FNLVIADARYL----------------PVLR   55
paDim1     59  ALAERLRGI-------APPNVVIIVGDALEV----------------EWP-   85
csDim1     51  RLYGEASASLH------CEGLELRRGDGFAV----------------E-D-   77
seKsgA     62  DLIGQLQQRFGQ-----AENFCLLEGDILQLDWTAAIA---------DRP-   97
bKsgA      81  RLLPILKDTLSPY-----ENVTVIHQDVLKADVKSVIE-----------EQ  115
mtuKsgA    89  LLASRLQQTVAEHSHSEVHRLTVVNRDVLALRRE-------------DLA-  125
ttKsgA     79  RLRPVLEETLSGLP------VRLVFQDALLYPWE-------------EVP-  109
bfKsgA     65  ESVAYLREAYPSL------EDNIIEDDFLKM----------------NLQR   93
ctKsgA     79  MFEESLSQLPMDIE--ITDACKYPLTSLED---K-------------GWK-  110
bbKsgA     88  KYSEILNEKFGKL-----KNFKLIKGDFLKKYKN-------------ENQ-  119
ecKsgA     70  DLAARLQTHPF-----LGPKLTIYQQDAMTF----------------NFGE   99

cons      154                            .                         204
```

```
atDim1     119  ---RF---DICVANIPYQISSPLTFKLL-FH-------P--TSFRCAVIMYQ  154
ddDim1     120  ---YF---DVCVANVPYQISSPLTFKLL-AH-------R--PIFRTAVLMFQ  155
lbDim1     161  ---YF---DKCVANVPYAISSALVFKLL-KT-------P---TFKCAVLMFQ  195
glDim1     101  ---PF---DLCVSNCPYNISSGIVFRLL-EI-------Q--PLPRKFVLMFQ  136
pvDim1     170  ---RF---DICTANIPYKISSPLIFKLI-AH-------R--PLFKCAVLMFQ  205
hsDim1     121  ---FF---DTCVANLPYQISSPFVFKLL-LH-------R--PFFRCAILMFQ  156
scDim1     121  ---YF---DICISNTPYQISSPLVFKLI-NQ-------P--RPPRVSILMFQ  156
atPFC1     175  S--AL---AKVVSNLPFNISTDVVKLLL-PM-------G--DIFSKVVLLLQ  211
dmTFB1     134  -------RIHLIGNLPFAISTRLLINWL-DDLAARRGAFRRIDTCMTLTFQ  176
ceTFB1     131  SNLPA---MHVIGNLPFNIASPLIIKYL-RDMSYRRGVWQYGRVPLTLTFQ  177
hsTFB1     134  ---PN---VHIIGNLPFSVSTPLIIKWL-ENISCRDGPFVYGRTQMTLTFQ  177
mkDim1     107  ---DV---NKVVANIPYNISSPITFKLL-EL-----------DIDVAVLTYQ  140
mthDim1     84  ---EY---NKVVSNLPYHISTKITLRLL-RN-----------PFDLMVLMYQ  117
hwDim1     129  ---AF---TTCISNLPYGISSEILFELL-PA-------D------CPLIVMVQ  161
mmaDim1     91  ---PF---EIVVANLPYSVSSKITFRLL-EI-----------GFEVAVLMYQ  124
mjDim1      93  L--DF---NKVVANLPYQISSPITFKLI-KR-----------GFDLAVLMYQ  127
phDim1     125  ---KF---NKVVSNIPYKISSPFTFKLL-KT-----------DFERAVVMYQ  158
msDim1     105  ---KF---DKVVSNLPYQISSPVTFKLL-EY-------P-----FKKAILMYQ  138
ptDim1      88  ---AY---DKIIGNIPYNISSQIIFKLY-DF-------D-----FKLALLMVQ  121
afDim1      93  ---YF---TKFVANIPYKISSPLTFKLL-KT-----------DFRLAVVMYQ  126
apDim1     118  ---QA---PLVFSNTPFNLSPAIVEALAVN----------NRVAAAVLGVQ  152
ssDim1      56  ---------GQLVSSLPYQITSDFFKEVI-KL----------NNIRKLTLILQ   88
paDim1      86  ---RA---DFFVSNVPYSITSPLLFKLI-RH-------R------LPAVLTIQ  118
csDim1      78  ---GF---DVFVSNLPYSQSRRAVEWLV-QR-------D-----FARGIVTVQ  111
seKsgA      98  ---RFANPSKVVANIPYNITGPILQSLL-GTIAQ-PRR--PAFERLVLLVQ  141
bKsgA      116  FQ-DC-DEIMVVANLPYYVTTPIIMKLL-EE-------H--LPLKGIVVMLQ  155
mtuKsgA    126  ---AA--PTAVVANLPYNVAVPALLHLL-VE-------F--PSIRVVTVMVQ  162
ttKsgA     110  ---QG---SLLVANLPYHIATPLVTRLL-KT-------G---RFARLVFLVQ  144
bfKsgA      94  LFD--GHPFVLTGNYPYNISSQIFFKML-DN-------K--DLIPCCTGMIQ  133
ctKsgA     111  ---GK---GRIVANLPYHITTPLLTKFFLEC-------P--YRWKTVTVMIQ  147
bbKsgA     120  ---NI---DKIFSNLPYNIASKVISKLI-EE----------NFLKEMVFTVQ  154
ecKsgA     100  LAEKMGQPLRVFGNLPYNISTPLMFHLF-SY-------T--DAIADMHFMLQ  141

cons       205                 ..  *:   :                          *  255
```

```
atDim1    155  REFAMRLVAQP-GDNLYCRLSVNTQLYARVSHLLKVGKNNFRPPPKVDSSV  204
ddDim1    156  KEFALRLGAKP-GDSLYCRLSVNTQLLSKVTHLMKVGKNNFLPPPKVESAV  205
lbDim1    196  REFALRVCAQP-GSEAYCRLSVNSQLLARCSHLMKISRNSFNPPPKVESSV  245
glDim1    137  LEFAQRLAAEP-GQDQYSRLTVNTKLLSKTKIIIRVSRNSFKPPPNVDSAV  186
pvDim1    206  KEFADRMLANV-GDSNYSRLTVNVKLFCKVVKICNVDRSSFNPPPKVDSVI  255
hsDim1    157  REFALRLVAKP-GDKLYCRLSINTQLLARVDHLMKVGKNNFRPPPKVESSV  206
scDim1    157  REFALRLLARP-GDSLYCRLSANVQMWANVTHIMKVGKNNFRPPPQVESSV  206
atPFC1    212  DEAALRLVEPALRTSEYRPINILINFYSEPEYNFRVPRENFFPQPKVDAAV  262
dmTFB1    177  QEVAERICAPV-GGEQRCRLSVMSQVWTEPVMKFTIPGKAFVPKPQVDVGV  226
ceTFB1    178  LEVAKRLCSPI-ACDTRSRISIMSQYVAEPKMVFQISGSCFVPRPQVDVGV  227
hsTFB1    178  KEVAERLAANT-GSKQRSRLSVMAQYLCNVRHIFTIPGQAFVPKPEVDVGV  227
mkDim1    141  REFAERMVAEP-GSKKYSRLTVMVNLLADVELLRGVPRRAFIPPPRVGSSV  190
mthDim1   118  REFVERMLASP-GSREYGRLSVNVSYYADVEVLETVPRSAFRPMPHVSSSV  167
hwDim1    162  REFGERMAADP-GTDAYGRLSVSAQHYATVEVVETVPPTAFAPEPAVDSAL  211
mmaDim1   125  KEFARRMVAPP-GTPNVGRLSVMVQTYASVKPLLELGPGSFRPQPAVRSWV  174
mjDim1    128  YEFAKRMVAKE-GTKDYGRLSVAVQSRADVEIVAKVPPSAFYPKPKVYSAI  177
phDim1    159  LEFALRMVAKP-GSRNYSRLSLMAQALGNVEIVMKIGKGAFYPRPKVDSAL  208
msDim1    139  LEFAKRMQAKP-DTHEYSRLSVALSYRADTKIIDTLPPEAFIPKPKIKSAV  188
ptDim1    122  REFAERLVASP-GNKNYSRLSASSKLRFDIKKVMDVSRKNFYPVPEVDSSI  171
afDim1    127  REFAERLCGED------NRLGVISKTYCKAEILEIVKPSSFNPPPKVESAI  171
apDim1    153  YEVARRMTARP-GSRDYSRLSVLVSLVFHAELAGVVRPQAYYPRPQVLTAV  202
ssDim1     89  KDFVDKIF----NDSTY--ISFLLNYIYNIQIKDIIPPSCFSPRPKVYSII  133
paDim1    119  REVAERLVARP-GSEDYGRLTVAVQCFYDVEILRVLPPYVFDPPPKVYSAV  168
csDim1    112  KEFAAKLMEAD-P-RRRRAIGVLAGHCFEMRVLFPVARSCFDPPPRVDSVV  160
seKsgA    142  QEVADRLCATP-GQRAYGALSVRVQYLASCERVCAVPPKSFSPPPKVQSTV  191
bKsgA     156  KEVAERMAADP-SSKEYGSLSIAVQFYTEAKTVMIVPKTVFVPQPNVDSAV  205
mtuKsgA   163  AEVAERLAAEP-GSKEYGVPSVKLRFFGRVRRCGMVSPTVFWPIPRVYSGL  212
ttKsgA    145  KEVAERMTARP-KTPAYGVLTLRVAHHAVAERLFDLPPGAFFPPPKVWSSL  194
bfKsgA    134  KEVAERIAAGP-GSKTYGILSVLIQAWYRVEYLFTVNEQVFNPPPKVKSAV  183
ctKsgA    148  DEVARRITAKP-GDKDYGSLTVFLSFFADVQYAFKVSPNCFYPKPSVHSAV  197
bbKsgA    155  KELADRITAKI-NSKNYSSFTVLVQSHFKVIKILDIGENNFYAPAKVKSTT  204
ecKsgA    142  KEVVNRLVAGP-NSKAYGRLSVMAQYYCNVIPVLEVPPSAFTPPPKVDSAV  191

cons      256   :   ::                            :    : * * :      306
```

```
atDim1     205 VRIEPRRP--GPQ--VNKKEWDGFLRVCFIRKNKTLGSIFKQKSVLSMLEK 251
ddDim1     206 VRIEPFNP--PPP--INFVEWDGLVKLCFSRKNKTLSGIFRVSSVIETLNQ 252
lbDim1     246 IRLDPKCP--PPD--VDFEEWDGLVKMLFNRKNKKASSIFRTKVAVQALYD 292
glDim1     187 VEMYPVGP--PPG--LDLDEFNGLTRILFSKKNKTLGAIFKTKSLLDDLAN 233
pvDim1     256 LKLIPKEN--NFF--INFDEWDNLLRICFSRKRKTLHAIFKRNAVLNMLEH 302
hsDim1     207 VRIEPKNP--PPP--INFQEWDGLVRITFVRKNKTLSAAFKSSAVQQLLEK 253
scDim1     207 VRLEIKNP--RPQ--VDYNEWDGLLRIVFVRKNRTISAGFKSTTVMDILEK 253
atPFC1     263 VTFKLKHPRDYP-DVSSTKNFFSLVNSAFNGKRKMLRKSLQHISSS----- 307
dmTFB1     227 VKLIPLKRPK---TQLPFHLVERVVRHIFSMRQKYCRRGYGTLLPPEDR-- 272
ceTFB1     228 VRFVPRKTPL---VNTSFEVLEKVCRQVFHYRQKYVTKGLKTLYPEELE-- 273
hsTFB1     228 VHFTPLIQPK---IEQPFKLVEKVVQNVFQFRRKYCHRGLRMLFPEAQR-- 273
mkDim1     191 VRLTPKSEEERPD--VDPDTLESVCRALFQHKNKTVRNALLLSAHEWA--- 236
mthDim1    168 VRLRPRRDRE--L--VDELIFSSISRDLFTKRRKKVKNALASIGVA----- 209
hwDim1     212 IRAEPRDPDY--T-VTDETFFLRFVKAVFTQRRKTVRNAIRNTAHISMLD- 258
mmaDim1    175 VRITPHEPPY-PI--ADRRVYADVVRVLFSHRRKTVRKGLRSGKDAFS--- 219
mjDim1     178 VKIKPNKGKY--H-IENENFFDDFLRAIFQHRNKSVRKALIDSSKELN--- 222
phDim1     209 VLIEPRKDKI--V--LN----ENLVKALFQHRRKTVPRALKDSIHMLG--- 248
msDim1     189 IELIPKNNKPI-------DKLLDNTIRALFQHRNKKAKKALIQSAHELG--- 230
ptDim1     172 IIIKKNNK--KIP--CNP---DDIIKMAFSMKRKKISSIFKNY-------- 207
afDim1     172 VRIVPEPEV--FV--ENRELFEKFVTFAFSMRRKRMGKIVQEFRKRYG--- 215
apDim1     203 VTLRRRRRWR---SL--YARALELAGCAFTQRNKKASKVLRRCLEAAGCA- 247
ssDim1     134 TIFNRIREYD---------KEVDSILSCISRYRNKTLRKASKLCGFSSNN-- 174
paDim1     169 VRLMPKAP--CVD---NFDEFEKFSAWLFSARRKTLRRLKLADSTKRVYQL 214
csDim1     161 VELAKRRT--IPG-------GVVRAVNGIFSYRRKTMRGIMKQFGREG---- 199
seKsgA     192 ICLKPRPWPQ-VC--NNPGRLEKLLNQGFSAKRKMLRNNLKSLYS------- 233
bKsgA      206 IRLILRDGPAVD--VENESFFFQLIKASFAQRRKTLLNNLVNNLPEGK--- 251
mtuKsgA    213 VRIDRYETSPWPTDDAFRRRVFELVDIAFAQRRKTSRNAFVQWAGSG---- 259
ttKsgA     195 VRLTPTGAPD--------DPGLFRLVEAAFGKRRKTLLNALAAAGYP------ 233
bfKsgA     184 IRMTRNETQE---LGCDPKLFKQIVKTTFNQRRKTLRNSIKPILGKDCP-- 229
ctKsgA     198 VHMRVHEQFALAD--SEIEEFFTLTRAAFGQRRKLLANSLKNLYP------ 240
bbKsgA     205 LKLIPKKNN-----IKNFKEFNKLVRTVFSNRRKKLKNTIINFITNK---- 246
ecKsgA     192 VRLVPHATMPHPV--KDVRVLSRITTEAFNQRRKTIRNSLGNLFSVEVLTG 240

cons       307                              :.:                      357
```

```
atDim1     252  NFKTLQA--------------VLASLQNNGEPALNTTSMDLGDQSMGMEDD  288
ddDim1     253  NYK------------------TYCALEGKMNT-----------D---G---  268
lbDim1     293  KYVSYRRM--------ESGQPVGSAASSSIVD-----------ASTPM---  321
glDim1     234  NIARLDEISRLHKPAQHEGSNPSHSASSIGAK-----------PSV-L---  269
pvDim1     303  NYK------------------NFCTFNKIVPV-----------NF------  318
hsDim1     254  NYR------------------IHCSVHNIIIP-----------E-------  268
scDim1     254  NYK------------------TFLAMNNEMVD-----------DTKGS---  272
atPFC1     308  ---------------------------------------------------  307
dmTFB1     273  ---------------------------------------------------  272
ceTFB1     274  ---------------------------------------------------  273
hsTFB1     274  ---------------------------------------------------  273
mkDim1     237  ---------------------------------------------------  236
mthDim1    210  ---------------------------------------------------  209
hwDim1     259  ---------------------------------------------------  258
mmaDim1    220  ---------------------------------------------------  219
mjDim1     223  ---------------------------------------------------  222
phDim1     249  ---------------------------------------------------  248
msDim1     231  ---------------------------------------------------  230
ptDim1     208  ---------------------------------------------------  207
afDim1     216  ---------------------VEL---------------------------  218
apDim1     248  ---------------------------------------------------  247
ssDim1     175  ---------------------------------------------------  174
paDim1     215  TLEE-----------------------------------------------  218
csDim1     200  ---------------------------------------------------  199
seKsgA     234  ---------------------------------------------------  233
bKsgA      252  ---------------------------------------------------  251
mtuKsgA    260  ---------------------------------------------------  259
ttKsgA     234  ---------------------------------------------------  233
bfKsgA     230  ---------------------------------------------------  229
ctKsgA     241  ---------------------------------------------------  240
bbKsgA     247  ---------------------------------------------------  246
ecKsgA     241  ---------------------------------------------------  240

cons       358                                                       408
```

```
atDim1    289 DNEMDDDDMEMDEGEGDGGETSEFKEKVMNVLKEGG--------FEEK-RS 330
ddDim1    269 -------------------SDEQMKELIIKTLTDND--------FLDS-RS 291
lbDim1    322 -------------------SLEQFRVLLDSVIADPM--------F-ET-RS 343
glDim1    270 -------------------TGVQLRERIAEALAEAN--------LTDT-RP 292
pvDim1    319 ----------------------PFKKYCLDTLKELD--------MTEC-RS 338
hsDim1    269 --------------------DFSIADKIQQILTSTG--------FSDK-RA 290
scDim1    273 -------------------MHDVVKEKIDTVLKETD--------LGDK-RA 295
atPFC1    308 -------------------------PDIEKALGVAG--------LPATSRP 325
dmTFB1    273 ------------------------EEVAEKLFQRAE--------VQDTLRP 291
ceTFB1    274 ------------------------DELSDDLLKKCR--------IDPTTTS 292
hsTFB1    274 ------------------------LESTGRLLELAD--------IDPTLRP 292
mkDim1    237 ----------------------TDREQAREVLEELPE------DLLS-ERP 258
mthDim1   210 -------------------------DDVMLDLDPS---------ILD-ARP 225
hwDim1    259 --------------------------TPDAVVNAADE------ALLS-QRA 276
mmaDim1   220 ------------------------PEAIGRTIASLPD------DLLQ-RRP 239
mjDim1    223 ----------------------YNKDEMKKILEDFLNTNSEIKNLIN-EKV 250
phDim1    249 ----------------------VSKDEIRGIINNVP--------HSNK-RV 268
msDim1    231 ----------------------VDKKVLKQKLSNVTN------DLFE-EKV 252
ptDim1    208 ---------------------------------DGP--------LKY-KRP 216
afDim1    219 ---------------------------------------AVNKNLAE-KRP 229
apDim1    248 -------------------------------------PPPWLDSLGD-ARV 260
ssDim1    175 -----------------------------------------------DLKV 178
paDim1    219 --------------------------------------------------- 218
csDim1    200 ----------------------------------------------GPGRL 204
seKsgA    234 ------------------------SEQIEAAFAAHQ--------IAPEARA 252
bKsgA     252 ----------------------AQKSTIEQVLEETN--------IDGKRRG 272
mtuKsgA   260 -------------------------SESANRLLAAS--------IDPARRG 277
ttKsgA    234 ------------------------KARVEEALRALG--------LPPRVRA 252
bfKsgA    230 ---------------------------------------LTEDALFN-KRP 240
ctKsgA    241 ------------------------KDKVFQVLEQLG--------FSEKTRP 259
bbKsgA    247 -------------------------ATLRENFL---------KEYLD-KRP 262
ecKsgA    241 ----------------------------------MG--------IDPAMRA 249

cons      409                                                     459
```

```
atDim1     331  SKLSQQEFLYLLSLFNKSGIHF-----------------------------  352
ddDim1     292  SKLDINDFLKLLNKFHETGIHF-----------------------------  313
lbDim1     344  RMLDEEALMTMLAHFIKHGIHF-----------------------------  365
glDim1     293  NHMAIPDLLSLMCALAAKGVRFT----------------------------  315
pvDim1     339  VSLDENDFLKLLLKFNKKGIHFFNISNVGSSRAANIVLNE--EGDVRGDGG  387
hsDim1     291  RSMDIDDFIRLLHGFNAEGIHF-----------------------------  312
scDim1     296  GKCDQNDFLRLLYAFHQVGIHF-----------------------------  317
atPFC1     326  EELTLDDFVKLHNVIARE---------------------------------  343
dmTFB1     292  FELTVEQCLRLAEVYSEHLVTRPEVAAYDYR--------------------  322
ceTFB1     293  IRLGIEQFADLAEGYNEQCIRYPGLFLYDYTNKLHNLEDLSKEPNALPPPV  343
hsTFB1     293  RQLSISHFKSLCDVYRKMCDEDPQLFAYNFREELKRRKSK-----------  332
mkDim1     259  LHLPPERVAELAAAIESAL--------------------------------  277
mthDim1    226  EDLGVDEFVQIARVVSEHRSRDS----------------------------  248
hwDim1     277  GDLTPSEFAELATIAAEKG--------------------------------  295
mmaDim1    240  EDLTLEEFALIANKMSGG---------------------------------  257
mjDim1     251  FKLSVKDIVNLSNEFYRFLQNR---------------------GR------  274
phDim1     269  FQLYPEEVKDIEEYLKKHGII------------------------------  289
msDim1     253  FKLTPTQIKEISLILEGN---------------------------------  270
ptDim1     217  GDLSPEDFIDLCCYLFPERCQG-----------------------------  238
afDim1     230  EELGARKFAEIVVG-------------------------------------  243
apDim1     261  WMLRPEDFVGLAEACRG----------------------------------  277
ssDim1     179  REFKPWQVLELLNSVG-----------------------------------  194
paDim1     219  ----------LVELFKR---HK-----------------------------  227
csDim1     205  DDLSGDEIVGVAAEIA-----------------------------------  220
seKsgA     253  ETLSIDQWIGLCTDLGDPTDSAI----------------------------  275
bKsgA      273  ESLSIEEFAALSNGLYKAL--------------------------------  291
mtuKsgA    278  ETLSIDDFVRLLRRSGG----------------------------------  294
ttKsgA     253  EELDLEAFRRLREGLEGA---------------------------------  270
bfKsgA     241  EQLSVQEFIHLTNQVEQAL---------------KVPIEPV----------  266
ctKsgA     260  ETIFLEEYLKIFHLLKDI---------------------------------  277
bbKsgA     263  ENISVEEFIQISNTLNAYH--------------------------------  281
ecKsgA     250  ENISVAQYCQMANYLAENAPLQE----------------------------  272

cons       460            :                                         510
```

```
atDim1     353  -----------------------------T  353
ddDim1     314  -----------------------------K  314
lbDim1     366  -----------------------------I  366
glDim1     316  -----------------------------N  316
pvDim1     388  VGGAGGAGSDSSDDSDSDGYDDVDSIHEKV  417
hsDim1     313  -----------------------------S  313
scDim1     318  -----------------------------S  318
atPFC1     344  ------------------------------  343
dmTFB1     323  ----------------------APKNVEVL  330
ceTFB1     344  PIFAPAPTIDSADNTW------SLKNFNCS  367
hsTFB1     333  ---------------NEEKEEDD-AENYRL  346
mkDim1     278  -----------------------------G  278
mthDim1    249  -----------------------------L  249
hwDim1     296  -----------------------------K  296
mmaDim1    258  ------------------------------  257
mjDim1     275  -----------------------------L  275
phDim1     290  -----------------------------S  290
msDim1     271  -----------------------------L  271
ptDim1     239  -----------------------------S  239
afDim1     244  -----------------------------E  244
apDim1     278  ------------------------------  277
ssDim1     195  ------------------------------  194
paDim1     228  -----------------------------A  228
csDim1     221  -----------------------------R  221
seKsgA     276  --------------------------NPTA  279
bKsgA      292  -----------------------------F  292
mtuKsgA    295  SDEATSTGRDARAP-------DISGHASAS  317
ttKsgA     271  -----------------------------V  271
bfKsgA     267  ------------------------SQIENP  272
ctKsgA     278  ------------------------------  277
bbKsgA     282  ------------------------------  281
ecKsgA     273  -----------------------------S  273

cons       511                                  540
```
