# Supplementary material for: Sequence and structural evolution of the KsgA/Dim1 methyltransferase family
Source: BMC Res Notes. 2008 Oct 29;1:108. doi: 10.1186/1756-0500-1-108 (PMC2614427; doi:10.1186/1756-0500-1-108)
Supplement: Additional file 2 — Sequence alignment of mtTFB, mtTFB1, and mtTFB2 proteins. The structure-based sequence alignment was perfomed using the program Expresso [18]. The structure 1I4W[11] was used for the alignment. Organisms represented are Caenorhabditis elegans (ce), Homo sapiens (hs), Drosophila melanogaster (dm), Anopheles gambiae (ag), Apis mellifera (am), Xenopus laevis (xl), Takifugu rubripes (tr), Ciona intestinalis (ci), Rattus norvegicus (rn), Pan troglodytes (pt), Mus musculus (mmu), Bos taurus (bt), Tetraodon nigroviridis (tn), Saccharomyces cerevisiae (sc), Schizosaccharomyces pombe (sp), Kluyveromyces lactis (kl), Eremothecium gossypii (eg), Candida albicans (ca), Dictyostelium discoideum (dd), Trypanosoma brucei (tb), and Leishmania major (lm). Accession numbers for each sequence are found in Additional file 4. [file 1756-0500-1-108-S2.pdf]

T-COFFEE, Version_5.31Fri Oct 26 17:01:36 2007
Cedric Notredame
CPU TIME:67 sec.
SCORE=27
*
BAD AVG GOOD
*

ceTFB1	:	33
hsTFB1	:	33
dmTFB1	:	29
agTFB1	:	33
amTFB1	:	33
xlTFB1	:	33
trTFB1	:	34
ciTFB1	:	31
hsTFB2	:	30
rnTFB2	:	29
dmTFB2	:	19
ptTFB2	:	31
mmuTFB2	:	29
btTFB2	:	29
trTFB2	:	28
tnTFB2	:	27
scTFB	:	18
spTFB	:	19
klTFB	:	15
egTFB	:	19
caTFB	:	19
ddTFB	:	31
tbTFB	:	27
lmTFB	:	27
cons	:	27

```
ceTFB1    1  ---------------------------------------------------   0
hsTFB1    1  ---------------------------------------------------   0
dmTFB1    1  MALVTTLSLEESLNSRICVRSSENGARSGIAENAVFPLSSNYALCNAITEK  51
agTFB1    1  ---------------------------------------------------   0
amTFB1    1  ---------------------------------------------------   0
xlTFB1    1  ---------------------------------------------------   0
trTFB1    1  ---------------------------------------------------   0
ciTFB1    1  -------------------------------------------MKISHRLL   8
hsTFB2    1  ---------------------------------------------------   0
rnTFB2    1  ---------------------------------------------------   0
dmTFB2    1  ---------------------------------------------------   0
ptTFB2    1  ---------------------------------------------------   0
mmuTFB2   1  ---------------------------------------------------   0
btTFB2    1  ---------------------------------------------------   0
trTFB2    1  ---------------------------------------------------   0
tnTFB2    1  -------MYRKGFKWSSCPRC---------------PFILYTQLQTSIMST  29
scTFB     1  ---------------------------------------------------   0
spTFB     1  ---------------------------------------------------   0
klTFB     1  ---------------------------------------------------   0
egTFB     1  ---------------------------------------------------   0
caTFB     1  MRILTKAR------------------------------AFNPHLA------  15
ddTFB     1  ---------------------------------------------------   0
tbTFB     1  -------MSRRFLNRSQ----------------------------------  10
lmTFB     1  ---------------------------------------------------   0

cons      1                                                        51

ceTFB1    1  ---------------------------------------------------   0
hsTFB1    1  ---------------------------------------------------   0
dmTFB1   52  LLWLTCRQVVAHCAWKSALRCRTSRRTRATNENFIILKSARKARKKGRLAA 102
agTFB1    1  ---------------------------------------------------   0
amTFB1    1  ---------------------------------------------------   0
xlTFB1    1  ---------------------------------------------------   0
trTFB1    1  ---------------------------------------------------   0
ciTFB1    9  NK-------------------------------------------------  10
hsTFB2    1  ---------------------------------------------------   0
rnTFB2    1  ---------------------------------------------------   0
dmTFB2    1  ---------------------------------------------------   0
ptTFB2    1  ---------------------------------------------------   0
mmuTFB2   1  ---------------------------------------------------   0
btTFB2    1  ---------------------------------------------------   0
trTFB2    1  ---------------------------------------------------   0
tnTFB2   30  KMCMVVELMRSACC--HTVRSRSCLRRPTML--------------------  58
scTFB     1  ---------------------------------------------------   0
spTFB     1  ---------------------------------------------------   0
klTFB     1  ---------------------------------------------------   0
egTFB     1  ---------------------------------------------------   0
caTFB    16  ---------------------------------------------------  15
ddTFB     1  ---------------------------------------------------   0
tbTFB    11  --------VTSASA--ALLYSRRGVSKPSTD--------------------  31
lmTFB     1  ------------------MHGASGSSLLPQA--------------------  13

cons     52                                                       102
```

```
ceTFB1      1  ---------------------------------------------------   0
hsTFB1      1  ---------------------------------------------------   0
dmTFB1    103  FIMSASEQGPKI--KYGESAPKLDKAQLQFMKLIEEQNLDRVQKLKRIRRN 151
agTFB1      1  --MSASEGPSKVEFKIEESGRKLKKAEVDFMRLIEQQNLQRVQKLQRQRRN  49
amTFB1      1  ---------------------------------------------------   0
xlTFB1      1  ---------------------------------------------------   0
trTFB1      1  ---------------------------------------------------   0
ciTFB1     11  ---------------------------------------------------  10
hsTFB2      1  ---------------------------------------------------   0
rnTFB2      1  ---------------------------------------------------   0
dmTFB2      1  ---------------------------------------------------   0
ptTFB2      1  ---------------------------------------------------   0
mmuTFB2     1  ---------------------------------------------------   0
btTFB2      1  ---------------------------------------------------   0
trTFB2      1  ---------------------------------------------------   0
tnTFB2     59  ---------------------------------------------------  58
scTFB       1  ---------------------------------------------------   0
spTFB       1  ---------------------------------------------------   0
klTFB       1  ---------------------------------------------------   0
egTFB       1  ---------------------------------------------------   0
caTFB      16  ---------------------------------------------------  15
ddTFB       1  ---------------------------------------------------   0
tbTFB      32  ---------------------------------------------------  31
lmTFB      14  ---------------------------------------------------  13

cons      103                                                      153

ceTFB1      1  ----------------------------------------------MASAS   5
hsTFB1      1  -----------------------------------MAA------SGKLSTC  10
dmTFB1    152  NLLTAGALGVSVLAIYGYSIFSVQQEKFLDDFEEPKKMAQPSA-RVLQSGM 201
agTFB1     50  NKLTGIALGGTVLGIYLYSMLSVKQEKFLDDFEEPMSASTKISKALTSTGI 100
amTFB1      1  -----------------------------------------------MSTI   4
xlTFB1      1  -----------------------------------MAT------PGALAKF  10
trTFB1      1  -----------------------------------MSA------SQKLASL  10
ciTFB1     11  -------------------------------FRPKLQL------NHDLPTQ  24
hsTFB2      1  ---------------------------------------------MWIPVV   6
rnTFB2      1  ---------------------------------------------MRGLAM   6
dmTFB2      1  ---------------------------------------------------   0
ptTFB2      1  ---------------------------------------------MWIPVV   6
mmuTFB2     1  ---------------------------------------------MRGPAM   6
btTFB2      1  ---------------------------------------------MWVPGA   6
trTFB2      1  ---------------------------------------------------   0
tnTFB2     59  ----------------------------------------SMA-PSCLSAV  68
scTFB       1  ---------------------------------------------------   0
spTFB       1  --------------------------------------------------M   1
klTFB       1  ------------------------------------------------MTK   3
egTFB       1  ---------------------------------------------------   0
caTFB      16  ---------------------------------------------------  15
ddTFB       1  ------------------------------------------M-TIKNLTT   8
tbTFB      32  -----------------------------------------AA-APPLPPL  40
lmTFB      14  -----------------------------------------AA-PVALPPL  22

cons      154                                                      204
```

```
ceTFB1      6 RLPP----LPALRDFI-----------------------------------  17
hsTFB1     11 RLPP----LPTIREII-----------------------------------  22
dmTFB1    202 RLPP----MPTIRELV----------------------------------- 213
agTFB1    101 RLPP----LPTIRDLV----------------------------------- 112
amTFB1      5 RLPP----LPSIKDVL-----------------------------------  16
xlTFB1     11 RLPP----LPTIGEIV-----------------------------------  22
trTFB1     11 RLPP----LPTVGELI-----------------------------------  22
ciTFB1     25 AIPP----MPTSSELL-----------------------------------  36
hsTFB2      7 GLPR----RLRLSALAGAGRFCILG-----------SEAATRKHLPARNHC  42
rnTFB2      7 RLPP----RLALSVLAGRGPSCILG-----------SGAATRKDWQERNRR  42
dmTFB2      1 MLPL----RCSWS---------FAR-----------ANYSTKKELVTRYSG  27
ptTFB2      7 GLPR----RLRLSALAGAGRFCILG-----------SEAATRKHLPARNHC  42
mmuTFB2     7 RLPP----RIALSALA-RGPSCILG-----------SGAATRKDWQTRNRR  41
btTFB2      7 GIPS----RLTLSAFTRAARFCVLN-----------SGVARWKDVPAENCR  42
trTFB2      1 ---------------------------------------------------   0
tnTFB2     69 GLPGVHRRAYSLDPLSSGSGRTTLGSFTPPHSAERSSPSSPSSALTHRNLS 119
scTFB       1 MSVP----IPGIKDIS-----------------------------------  12
spTFB       2 KLPK-----ILYDAAAFG-------------------GPRSTGFV------  22
klTFB       4 SSFL----KSVLPLAN-----------------------------------  15
egTFB       1 --------------MSSASKF--------------SKQSFLEACL------  17
caTFB      16 ---------------------------------------------------  15
ddTFB       9 SLPP----MPKIQEII-----------------------------------  20
tbTFB      41 RCPGGPR----------------------------VKAEGLKQLF------  57
lmTFB      23 RCPGGPR----------------------------VKAEGIKQLY------  39

cons      205                                                     255

ceTFB1     18 ---------------HMYRLRAKKIL-----------SQNYLMDMNITRKI  42
hsTFB1     23 ---------------KLLRLQAANEL-----------SQNFLLDLRLTDKI  47
dmTFB1    214 ---------------KLYRLQARKQL-----------SQNFLMDERLTDKI 238
agTFB1    113 ---------------KLYQLRAIKQL-----------SQNFLMDERLTDKI 137
amTFB1     17 ---------------KIYRLRAMKEL-----------SQNFILNQNLADKI  41
xlTFB1     23 ---------------KLFNLRAEKQL-----------SQNFLLDLKLTDKI  47
trTFB1     23 ---------------KLYNLRAEKQL-----------SQNFLLDLKLTDKI  47
ciTFB1     37 ---------------KMYNVRARKQL-----------SQNFLLDPLITNRF  61
hsTFB2     43 GLSDSSPQLWPEPDFRNPPRKASKASLDFKRYVTDRR---------LAETL  84
rnTFB2     43 SFSDLYTQPLPDCDFEESSSWTHKSRSEPTRHIACKK--------SARNLV  85
dmTFB2     28 DFPE---------------KLLNRKQKVP--------THMYIANSEAAARI  55
ptTFB2     43 GLSDSSPQLWPEPDFRNPP---RKASLDFKRYVTDRR---------LAETL  81
mmuTFB2    42 GFSDFNIEPLPDSDLEESSPWTSRNRSEPTRHIACKK--------AARNLV  84
btTFB2     43 GLYDFHTQLKPDVEFGKLSSRLYKSRSE---------TKRYVTSPRVAETV  84
trTFB2      1 -------------------------------------MRHFIVNPDLAKLI  14
tnTFB2    120 AVAVQGQRRLCRYDFLDLGE----VEENVRRAQACKKIRHFIVDPDLAKLV 166
scTFB      13 ---------------KLKFFYGFKYLWNPT----------------VYNKI  32
spTFB      23 ---------------KILNLNGRSSY-----------KSSYLVNQNLMDEA  47
klTFB      16 ---------------KIHTSYGSQFEKNPK----------------VINQI  35
egTFB      18 ---------------AVKFSYGRTLLHSPT----------------AIELA  37
caTFB      16 ---------------EMFSNKIPYYY-----------SRAHMADPEACQKI  40
ddTFB      21 ---------------RIFGLSAKQQL-----------SQNFLIDKNITDKI  45
tbTFB      58 ---------------KVPHAGYLAKY-----------GQRFILNLKLTHQV  82
lmTFB      40 ---------------KVPHAGFLAKY-----------DQRFMLNLKLTHQL  64

cons      256                                                     306
```

```
ceTFB1    43  AK-HA------KVIEKDWVIEIGPGPGGITRAILEAG--ASRLDVVEIDNR   84
hsTFB1    48  VR-KA------GNLTNAYVYEVGPGPGGITRSILNAD--VAELLVVEKDTR   89
dmTFB1   239  VK-SA-----GRIDPRDLVLEVGPGPGGITRSILRRH--PQRLLLVEKDPR  281
agTFB1   138  VR-AA------GNIRDHYVLEVGPGPGGITRSIIRQN--PRHLVVVEKDRR  179
amTFB1    42  IK-KT------GNLNDCHVLEIGPGPGALTRSILKCQ--PKKLIVVEKDKR   83
xlTFB1    48  VR-RA------GNLQNAYVCEVGPGPGGITRSILNAG--VEELLVVEKDTR   89
trTFB1    48  VR-QA------GCLKDAHVCEVGPGPGGLTRSILNAG--AADLLVVEKDSR   89
ciTFB1    62  VLCGA------KDLAGHHVCEVGPGPGPITRSILQRK--PERLTVVEKDHR  104
hsTFB2    85  AQ---IYLGKP-SRPPHLLLECNPGPGILTQALLEAGA---KVVALESDKT  128
rnTFB2    86  R----DLLEHQ-NPSHQLILECNPGPGILTGALLKAGA---RVVAFESEKM  128
dmTFB2    56  NQ-Y--LEPHFQSSGCDTVMELNSGAGYFTRHLLDRESQFRRIILLESMDH  103
ptTFB2    82  AQ---IYLGKP-SRPPHLLLECNPGPGILTQALLEAGA---KVVALESDKT  125
mmuTFB2   85  R----DLLEHQ-NPSRQIILECNPGPGILTGALLKAGA---RVVAFESEKT  127
btTFB2    85  VR----VLRGK-RKAGQLILECNPGPGVLTRALLESGA---RVIALESDKN  127
trTFB2    15  T----QHLLP--ENAATIIFECNPGPGVLTRTLLNSGV--QKVVALEGDKV   57
tnTFB2   167  T----QHLQP--DNATTIIFDCNPGPGVLTRTLLNSGI--QKVVALEGEKF  209
scTFB     33  FD-KLDLTKTYKHPEELKVLDLYPGVGIQSAIFYNKYC--------PRQYS   74
spTFB     48  LV-KSNLLKEY-NSEKMTILEMAPGPGVTTTSLFNYFQ-PKSHVVLESREV   95
klTFB     36  LD-KLNLESYY-KSESLQILDIYAGPLIQSVILNERLK--------PKKHV   76
egTFB     38  YK-RLNLQEHY-DMSKVQVLELYPGTGLPSYIFHDI---------------   71
caTFB     41  LD-KLDLKSKY-DGSKLDIVDVNPGYGLFSTML-NYELKPRNHILIENKER   88
ddTFB     46  CK-KS------GGFDDCTVIEVGAGPGGLTRSLLTSG--AKKVIAVEMDPR   87
tbTFB     83  AA-LLSRTT--LRTPDKLLLELGPGAGALTRSLLTRP--CVGVLGIEQDER  128
lmTFB     65  VS-YLSRTT--LTTPDKVLVELGPGVGSLTRSLLTRP--CVGVLGIEVDER  110

cons     307                    : :  .*    :  :                   357

ceTFB1    85  FIPPLQHLAE---AADS---RMFIHHQDA--------LRTEIGDIWKN---  118
hsTFB1    90  FIPGLQMLSD---AAPG---KLRIVHGDV--------LTFKVE--------  118
dmTFB1   282  FGETLQLLKE---CASPLNIQFDIHYDDI--------LRFN----------  311
agTFB1   180  FMPTMEMLAEV---AQPFM-RMDIVQGDI--------LDYRVA--------  210
amTFB1    84  FEPTLEMLADAFETING---KMEIIFDDI--------MKINMS--------  115
xlTFB1    90  FIPGLKMLNE---ASGG---KVRTVHGDI--------LTYRMD--------  118
trTFB1    90  FIPGLKLLSE---AAPG---RVRIVHGDI--------LTYRMD--------  118
ciTFB1   105  FLPMLKYVAD---VSND---RMTIVHGDI--------LKYDLS--------  133
hsTFB2   129  FIPHLESLGK---NLDG---KLRVIHCDF--------FKLDPRS-------  158
rnTFB2   129  FIPHLESLRK---NADG---ELQVVHCDF--------FKIDPRY-------  158
dmTFB2   104  FMPKIQELHT---LYPE---RVKVRQGDF--------VNLWKLVYMDKMDG  140
ptTFB2   126  FIPHLESLGK---NLDG---KLRVIHCDF--------FKLDPRS-------  155
mmuTFB2  128  FIPHLEPLQR---NMDG---ELQVVHCDF--------FKMDPRY-------  157
btTFB2   128  FIPELKSLGN---SVNG---RLEVIYCDF--------FKLDPRN-------  157
trTFB2    58  FLSELQALEV---QLDG---QLEVVNCDF--------FKLDPIG-------   87
tnTFB2   210  FLPELQDLEI---QLDG---QLEVVHCDF--------FKLDPIG-------  239
scTFB     75  LLEKRSSLYK---FLNA---KFEGSPLQI--------LKRDPYD-------  104
spTFB     96  FSKPLQKLCT---LSDG---RIKWVHQDG--------YYW-----------  121
klTFB     77  LLEDRLKFVE---LYQA---TLKDHPSMV------N-YNKNPYK-------  107
egTFB     72  YKPKLQVLME---SKPA---YAKVIEQHLTLLDNIKLHKEDPYM-------  109
caTFB     89  CVTSLSSIIN---KLVE--ETGH--------NSNFTLYKKDSFI-------  119
ddTFB     88  FYPALKMLEE---SSGG---RMSLIMANM--------MDVDEA--------  116
tbTFB    129  FNGHLEQIRQ---YTSG---KFQWTNGDV--------LRINELEIVESLYA  165
lmTFB    111  FNPHLEQIRN---YTNN---KFQWVTADV--------LKVDELELLKSAFP  147

cons     358         .                                            408
```

```
ceTFB1    119 --------------------------------------------------- 118
hsTFB1    119 --------------------------------------------------- 118
dmTFB1    312 --------------------------------------------------- 311
agTFB1    211 --------------------------------------------------- 210
amTFB1    116 --------------------------------------------------- 115
xlTFB1    119 --------------------------------------------------- 118
trTFB1    119 --------------------------------------------------- 118
ciTFB1    134 --------------------------------------------------- 133
hsTFB2    159 --------------------------------------------------- 158
rnTFB2    159 --------------------------------------------------- 158
dmTFB2    141 --------------------------------------------------- 140
ptTFB2    156 --------------------------------------------------- 155
mmuTFB2   158 --------------------------------------------------- 157
btTFB2    158 --------------------------------------------------- 157
trTFB2     88 ---------------------------------------------------  87
tnTFB2    240 --------------------------------------------------- 239
scTFB     105 --------------------------------------------------- 104
spTFB     122 --------------------------------------------------- 121
klTFB     108 --------------------------------------------------- 107
egTFB     110 --------------------------------------------------- 109
caTFB     120 --------------------------------------------------- 119
ddTFB     117 --------------------------------------------------- 116
tbTFB     166 GFAQQHRRKPAADARERASSD----GNKSDGGEACSSGTKDSGCCTDDFYC 212
lmTFB     148 HFVKANIRRPPSPGQETWAANSAADPAASSGSQRVTF-TDDAGEN-DADGC 196

cons      409                                                     459

ceTFB1    119 ------------------------------------------ETARP---- 123
hsTFB1    119 ------------------------------------------KAFSE---- 123
dmTFB1    312 -------------------------------------------------IE 313
agTFB1    211 ------------------------------------------EAFPD---- 215
amTFB1    116 ------------------------------------------NLFPS---- 120
xlTFB1    119 ------------------------------------------RAFPK---- 123
trTFB1    119 ------------------------------------------RGFPR---- 123
ciTFB1    134 ------------------------------------------QCFPQ---- 138
hsTFB2    159 ------------------------------GGVIKPPAMSSRGLFKN---- 175
rnTFB2    159 ------------------------------QELVRPD-VNSHTIFQN---- 174
dmTFB2    141 -------------------------------------GSRVADLLSD---- 150
ptTFB2    156 ------------------------------GGVIKPPAMSSRGLFKN---- 172
mmuTFB2   158 ------------------------------QEVVRPD-VSSQAIFQN---- 173
btTFB2    158 ------------------------------HGMVTPPVMTSDMLFQY---- 174
trTFB2     88 ------------------------------SGNLKPPAMFTDKLFTD---- 104
tnTFB2    240 ------------------------------SGNLKPPAMFTDKLFTD---- 256
scTFB     105 ------------------------------WSTYSNL-IDEERIF------ 118
spTFB     122 -------------------------------QTYED--VYVSKVL------ 133
klTFB     108 ------------------------------WETFLEM-TNEDRVL------ 121
egTFB     110 ------------------------------WESFVSL-IDEKKIM------ 123
caTFB     120 ------------------------------WETYNDL-IDKDKLI------ 133
ddTFB     117 ------------------------------------------KLLRD---- 121
tbTFB     213 EDAPLRNVQREKILRKRFGKYAAFNHDTASGQNSGSAAPSSDVFGCAFSSE 263
lmTFB     197 EGAPLRSAQRERLLRQRRARQPYQHSDDTHCRGAEGAADPETA-----ASP 242

cons      460                                                     510
```

```
ceTFB1    124  ------ESVDWHDS----NLPAMHVIGNLPFNIASPLII-KYLRDMSYRRG  163
hsTFB1    124  ---SLK--RPWE-----DDPPNVHIIGNLPFSVSTPLII-KWLENISCRDG  163
dmTFB1    314  QHI-------------PDTSQRIHLIGNLPFAISTRLLI-NWLDDLAARRG  350
agTFB1    216  ---CPP--HDWMD----RKRAPVHLIGNLPFAISTRLLI-NWLRDMSLRTG  256
amTFB1    121  ---TEI--KAWT-----EKCPRIKLIGNLPFNVSTPLII-KLLHAISEKRD  160
xlTFB1    124  ---HLI--KSWD-----DEPPNVHIIGNLPFSVSTPLII-KWLEQVADRTG  163
trTFB1    124  ---DIS--KKWH-----EDPPNLHIIGNLPFSVSTPLII-KWLENIANQSG  163
ciTFB1    139  ---ELA--KDWH-----KASPPFIVFGNLPFNVSLPLIF-KWFEQISRKDG  178
hsTFB2    176  ---LGIEAVPWT------ADIPLKVVGMFPSRGEKRALW-KLAYDLYSCTS  216
rnTFB2    175  ---LGIKAVPWS------AGVPIKVFGILPNKHERRLLW-KILFDLYSCES  215
dmTFB2    151  -----VPQKAFT------DDINMLVFGAV---GSYPFFK-HLINSLIFQTS  186
ptTFB2    173  ---LGIEAVPWT------ADIPLKVIGMFPSRGEKRALW-KLAYDLYSCTS  213
mmuTFB2   174  ---LGIKAVPWS------AGVPIKVFGILPYKHERRILW-KILFDLYSCES  214
btTFB2    175  ---LGVKAHPWK------KGFPLKVVGILPAKTERNTLW-KILHDLYSCSS  215
trTFB2    105  ---LGISEASWT------DDIPVKVVGVLPMSNERGMLL-KMVYALFERLS  145
tnTFB2    257  ---LGISEASWT------DDIPVKVVGMLPLRNERGMLL-KMVYALFERLS  297
scTFB     119  -----VPEVQSSDH----INDKFLTVANVTGEGSEGLIM-QWLSCIGNKNW  159
spTFB     134  -----DPRIQTEEEQKLSPHRELLFFAHLPHGYAGLLFVSQILDFLSARDW  179
klTFB     122  -----TPSMQKRDH----IHNEFLIAANLTNKKGEQLYV-QYLQCIANQNW  162
egTFB     124  -----QPEVQTRDH----IHDSFIVMGNLTDKRGEQLYM-QYLQCIANKNW  164
caTFB     134  -----QPQIKSFDT----PHDELLILANWTGNKEESVLA-QWIKCCGHRNW  174
ddTFB     122  ---AGAETTNWK------DKSKVKIIGNLPFNVGTHLML-KWIRQIAPRQG  162
tbTFB     264  SAAFEVSDRWWS-----DGDAKLEVIANLPFNIITELLM-RYAVDCSRKQN  308
lmTFB     243  NAAFDVTNHRWS-----NGNAKVEVIANLPFEIITELLM-RYAADCSQHRG  287

cons      511                      .   .                 .             561

ceTFB1    164  VWQYGRVPLTLTFQLEVAKRLCSPIA--CDTRSRISIMSQYVAEPKMVFQI  212
hsTFB1    164  PFVYGRTQMTLTFQKEVAERLAANTG--SKQRSRLSVMAQYLCNVRHIFTI  212
dmTFB1    351  AFRRIDTCMTLTFQQEVAERICAPVG--GEQRCRLSVMSQVWTEPVMKFTI  399
agTFB1    257  AWSYGRASLTLTFQKEVAERIVAPIL--SDQRCRLSVMNQIWSTPELRFMI  305
amTFB1    161  AWTFGKTRMTLTFQKEVAERLIAQPL--DVQRCRLSVMAQAWTHPVLHFII  209
xlTFB1    164  PFTYGRTQMTLTFQQEVAERLTASTK--NKQRSRLSIMSQYLCNVKNCFTI  212
trTFB1    164  PFAYGRTRLTLTFQKEVAERLTASTG--SRQRSRLSIMAQYLCTIHSCFTI  212
ciTFB1    179  MFKLGRIPLVLTFQREVVERFLAQTG--DKQRCRLSVSAQNFCDIDYKFII  227
hsTFB2    217  IYKFGRIEVNMFIGEKEFQKLMADPGN-PDLYHVLSVIWQLACEIKVLHME  266
rnTFB2    216  IYRYGRVELNMFISEKEFRKLIATPKR-PDLYQVLGVLWQVACEIKFLHME  265
dmTFB2    187  LFNLGRCEMILAMPPPIYIHLTCNNEIGYLIYRSTSVLFQILFEHKFIAKV  237
ptTFB2    214  IYKFGRIEVNMFIGEKEFQKLMADPGN-PDLYHVLSVIWQLACEIKVLHME  263
mmuTFB2   215  IYRYGRVELNMFVSEKEFRKLIATPKR-PDLYQVMAVLWQVACDVKFLHME  264
btTFB2    216  VYKYGRAELNLFISEKECRKLTANPQT-PALYQSLSVLGQTACGIKVLCTE  265
trTFB2    146  IYRYGRIELNLFMSEKEYLKLSSQPGD-MMNYRASSVLWQMACDIELLHKE  195
tnTFB2    298  FYRYGRIELNLFISEKEYLKLTSRPGD-MMNYRAFSVLWQMACDIELLHKE  347
scTFB     160  LYRFGKVKMLLWMPSTTARKLLARPG--MHSRSKCSVVREAFTDTKLIAIS  208
spTFB     180  LGIFGRVRVLLWLPCSPTVTLLGSRG--FSKRSKTSVFREAFTDSRVLAAS  228
klTFB     163  MQRFGLVKMLVWIPQQTARKLFAPFS--NKDRNRLTLLSELATNTKLVATS  211
egTFB     165  MQRFGLVRMLFWVPQTTAIKLLSPCG--FKSRSRCSVITEAVTDTRLIATT  213
caTFB     175  LMKYGKVRMVIFAPSVSAMKFLGEPG--FKKRRRTGLKRDLYTDSRLIGVV  223
ddTFB     163  LYEFGRVPMYLMFQKELSDRICAQVG--SEEYSRLSVMVQQMCQPSIVYSI  211
tbTFB     309  LFVFGRVPLHVFTQQEVAECIIAPAG--SIHFSRLSVLCQCFFHTQLLRTF  357
lmTFB     288  LFAFGRVPIHVFTQREVAERILAPAG--SVQFSRLSVLCQCFFHVRLKQTF  336

cons      562          : .         :               : :             612
```

```
ceTFB1    213  SGS-CF----------------------------VPRPQVDVGVVRFV--P  232
hsTFB1    213  PGQA-----------------------------FVPKPEVDVGVVHFT--P  232
dmTFB1    400  PGKA-----------------------------FVPKPQVDVGVVKLI--P  419
agTFB1    306  SGRA-----------------------------FVPKPEVDVGVVTIV--P  325
amTFB1    210  PGTA-----------------------------FIPKPKVDVGLVTFV--P  229
xlTFB1    213  PGRA-----------------------------FIPKPKVDVGVVHLT--P  232
trTFB1    213  PGRA-----------------------------FVPKPEVDVGVVHFT--P  232
ciTFB1    228  AGGS-----------------------------FVPPPKVEVGVVKIV--P  247
hsTFB2    267  PWS-SF---DIYTRKGPLE----NPKRRELLD----QLQQKLYLIQMI--P  303
rnTFB2    266  PWS-SF---SVHAENGHLE----KSKHSESLN----LLKQNLYLVRMT--P  302
dmTFB2    238  PRE-DFL--PQQMAYSPTK----SSKLGKVQS----INPEYLYLVKFT--P  275
ptTFB2    264  PWS-SF---DIYTRKGPLE----NPKRRELLD----QLQQKLYLIQMT--P  300
mmuTFB2   265  PWS-SF---SVHTENGHLE----KSKHGESVN----LLKQNLYLVRMT--P  301
btTFB2    266  PSS-LF---DTYAIKGELE----KQRHRE-------SLEQNLCFVQLT--P  299
trTFB2    196  SWD-SF---VMSSRPR---------IRSTKSK----FPNDSLCLVRLR--P  227
tnTFB2    348  SWE-SF---VTSSRRS---------VRSPKNK----LPNDNLCLVRLR--P  379
scTFB     209  DANELK---GFDSQCIEEW----DPILFSAAE-IWPTKGKPIALVEMD--P  249
spTFB     229  ESTLQKLCMGYSKEAKENYQISPNPLLVSPTPITSEPHKEDLTLVEMCSKP  279
klTFB     212  ENS-VK---KFLPDCIEKF----DPVIIPSDN----KSPDDLSLVEIN--P  248
egTFB     214  PDNL-A---SFGPGVLDKH----DP-------LILPENKTDYALLEVL--P  247
caTFB     224  NSEKAP-GLGYDARVLVRD----QPVLLEPSS---AHRDEDYSVIEIS--P  264
ddTFB     212  PGT-AF----------------------------VPPPKVDASVVAIE--P  231
tbTFB     358  REMT-----------------------------YYPKTAVLGALITLQ--P  377
lmTFB     337  VDQT-----------------------------YYPRTEVEGAMLTLE--P  356

cons      613                                             .: .   *  663


ceTFB1    233  RKTPL----VNTSFEVLEKVCRQVFHYRQKYVTKGLKTLYPEE--LEDELS  277
hsTFB1    233  LIQPK----IEQPFKLVEKVVQNVFQFRRKYCHRGLRMLFPEA--QRLEST  277
dmTFB1    420  LKRPK----TQLPFHLVERVVRHIFSMRQKYCRRGYGTLLPPE--DREEVA  464
agTFB1    326  LQTPL----TQVHFDTVEKVVRHIFSMRQKYCRRGVANLYPPA--VREELT  370
amTFB1    230  LTIPR----TKHEFSIFEKVTRHIFSFRQKYGIRGVETLFPLE--YRTELA  274
xlTFB1    233  FVQPK----IEQPFKLVEKVVRCIFQFRRKYCHHGVSILFPEE--IRIQLT  277
trTFB1    233  LVKAQ----IQQPFKLVEKVVRNVFQFRRKHCHKGIEKLFPEA--CRPEMT  277
ciTFB1    248  KKTFD----INLPFKKIDYVVKHTMHRKSKYCKHSVKTMFPPK---RSDLV  291
hsTFB2    304  RQNLFTKNLTPMNYNIFFHLLKHCFGRRSATVIDHLRSLT------PLDAR  348
rnTFB2    303  RRTLFTENLSPLNYDMFFHLVKHCFGKRNAPIIHHLRSLS------TVDPI  347
dmTFB2    276  RRNLHELCQSQ-DLPALWFFIKQNYVSRRNRIIPNLEKWV------PGCGP  319
ptTFB2    301  RQNLFTKNLTPMNYNIFFHLLKHCFGRRSATVIDHLRSLT------PLDAR  345
mmuTFB2   302  RRTLFTENLSPLNYDIFFHLVKHCFGKRNAPIIRHLRSLS------TVDPI  346
btTFB2    300  HRNLFTGTLTPFNYDVFFHMLRQCFMKRNAKLIDHLPSLS------PIDAV  344
trTFB2    228  HANLFSAGLTSSNASTLLMMVKQCLAKRKVKLIDRLNLWS------PDSGS  272
tnTFB2    380  RADLFSAGLTPSNASTLLMMVKQCLAKRKVKLIDRLNLWS------PDSGS  424
scTFB     250  IDFD-------FDVDNWDYVTRHLMILKRTPLNTVMDSLGHGGQQYFNSRI  293
spTFB     280  QDKQ-------LSIPVFESIVRILLTCKATSLSKSIYYLGPGAETLLPS--  321
klTFB     249  RDHS-------IDLDHWDFVTQKLMILKSKPVEEMIEILGHGARDWFISRL  292
egTFB     248  LNHN-------MKLEYWDYCMQRLLVCKSTPLEDILEVLGHGASDFLKCRI  291
caTFB     265  GKY------TASQIENIEHFLSAIYLTN-KKLVDILPTLAPGAMYM-----  303
ddTFB     232  RISPLGD-EPVKDHHYFEFVCRELFSQRRKKLSNTIKTLGKDAESL-----  276
tbTFB     378  RAVPLLPGLDAATLIHFTDLLMRP-GQRGMTVYKALQQHV------PPEVA  421
lmTFB     357  RSVPLAHGLSASSLIHFTNLLMKP-GLRAATVHKSLSRFA------PAELV  400

cons      664                             .                        714
```

```
ceTFB1   278  DDLLKKCRIDPTTTS-----------------------------------I  293
hsTFB1   278  GRLLELADIDPTLRP-----------------------------------R  293
dmTFB1   465  EKLFQRAEVQDTLRP-----------------------------------F  480
agTFB1   371  EQTFKRADVDPLARS-----------------------------------F  386
amTFB1   275  QMMYKLSDLNPQTRP-----------------------------------V  290
xlTFB1   278  EQMLRLADVDPTLRP-----------------------------------T  293
trTFB1   278  QEVMQRADVDPALRP-----------------------------------T  293
ciTFB1   292  DEIFRKSGVDPLTRS-----------------------------------N  307
hsTFB2   349  -DILMQIGKQEDEKV-----------------------------------V  363
rnTFB2   348  -NILRQIRKRPGDTA-----------------------------------A  362
dmTFB2   320  -RLIINPKSSESVTPIYPDELPKKLPQYSCQSTTMSTRNYYPGINIYTQFG  369
ptTFB2   346  -DILMQIGKQEDEKV-----------------------------------V  360
mmuTFB2  347  -NILRQIRKNPGDTA-----------------------------------A  361
btTFB2   345  -HILKQIKKKKDVRV-----------------------------------V  359
trTFB2   273  -KLLAEMGMQEDILT-----------------------------------G  287
tnTFB2   425  -KLLAEMGMQEDILT-----------------------------------G  439
scTFB    294  -TDKDLLKKCPIDL------------------------------------T  307
spTFB    322  ---FTQCGINIDMPV-----------------------------------G  334
klTFB    293  --DPVLLKKKPYEL------------------------------------T  305
egTFB    292  --DPELLKKKPMQL------------------------------------T  304
caTFB    304  ---------------------AKDLPEEMLQKSS----------------Y  317
ddTFB    277  ----LGDDIDPKIRT-----------------------------------Q  288
tbTFB    422  QYMLQELRTDGALTV-----------------------------------L  437
lmTFB    401  QYMLQELRMDGAMTV-----------------------------------L  416

cons     715                                                      765


ceTFB1   294  RLGIEQFADLAEGYNEQCIRY------------------------------  314
hsTFB1   294  QLSISHFKSLCDVYRKMCDED------------------------------  314
dmTFB1   481  ELTVEQCLRLAEVYSEHLVTR------------------------------  501
agTFB1   387  QLSVAECLRIVEAYDGLVRER------------------------------  407
amTFB1   291  ELTIENIDNL-----------------------------------------  300
xlTFB1   294  ELTMTHFKKLCNVYREMCDQN------------------------------  314
trTFB1   294  ELTIPQIRALADAYAHLCTLE------------------------------  314
ciTFB1   308  ELENLHFRDLCYAYEEIADRI------------------------------  328
hsTFB2   364  NMHPQDFKTLFETIERSKDCA------------------------------  384
rnTFB2   363  KMYPHDFKRLFETIERSEDSV------------------------------  383
dmTFB2   370  DLLPSQILTLFSQFRQWPEYG------------------------------  390
ptTFB2   361  NMHPQDFKTLFETIERSKDCA------------------------------  381
mmuTFB2  362  RMYPHDFKKLFETIEQSEDSV------------------------------  382
btTFB2   360  DMYPKDFLRLFETIECSKDDT------------------------------  380
trTFB2   288  HVHPEEYLQLFQMMDKSQEFT------------------------------  308
tnTFB2   440  HVYPEEYLRLFQLMDKSQEFT------------------------------  460
scTFB    308  NDEFIYLTKLFMEWPFKPDIL------------------------------  328
spTFB    335  LLSAADFLTISKIIQKYPFKH------------------------------  355
klTFB    306  YLEIDEIAKVFALWPFKPSLL------------------------------  326
egTFB    305  NQEFTKIASLYALWPFKPSI-------------------------------  324
caTFB    318  EFTCEDIIKLSEAYENW----------------------------------  334
ddTFB    289  NLQIEQFVKITNRYIEFPNKIAIDHLEFENDGKTKRKEEKLKERVNKINTR  339
tbTFB    438  DLTTEEVCKLATLWHRFLEAX------------------------------  458
lmTFB    417  DLSVVEVTRLACLWQQFVTMS------------------------------  437

cons     766           :                                          816
```

```
ceTFB1    315 ----------------------------------------PGLFLYDYTNK 325
hsTFB1    315 ----------------------------------------PQLFAYNFREE 325
dmTFB1    502 --------------------------------------------------- 501
agTFB1    408 ----------------------------------------PEIAAYDY--- 415
amTFB1    301 --------------------------------------------------- 300
xlTFB1    315 ----------------------------------------PHLFSYNYREE 325
trTFB1    315 ----------------------------------------PDLQSYEFREE 325
ciTFB1    329 ----------------------------------------PNLRKVDTYGE 339
hsTFB2    385 --------------------------------------------------- 384
rnTFB2    384 --------------------------------------------------- 383
dmTFB2    391 --------------------------------------------------- 390
ptTFB2    382 --------------------------------------------------- 381
mmuTFB2   383 --------------------------------------------------- 382
btTFB2    381 --------------------------------------------------- 380
trTFB2    309 --------------------------------------------------- 308
tnTFB2    461 --------------------------------------------------- 460
scTFB     329 --------------------------------------------------- 328
spTFB     356 --------------------------------------------------- 355
klTFB     327 --------------------------------------------------- 326
egTFB     325 --------------------------------------------------- 324
caTFB     335 --------------------------------------------------- 334
ddTFB     340 IEKEKKKQELILEEKKLKKEQREKEKQELKENFEKQEIEIQNLTKFDLEDK 390
tbTFB     459 --------------------------------------------------- 458
lmTFB     438 --------------------------------------------------- 437

cons      817                                                     867

ceTFB1    326 LHNLEDLSKEP-----------------------NALPPPVPIFAPAPTID 353
hsTFB1    326 LKRRKSKNEE----------------------------------------- 335
dmTFB1    502 --------------PEVAAYDYR---------------------------- 510
agTFB1    416 ----------------------RAPK------------------------- 419
amTFB1    301 -----------------ISYHYLIQK------------------------- 309
xlTFB1    326 LRMKKLQGKS----------------------------------------- 335
trTFB1    326 LRL------------------------------------------------ 328
ciTFB1    340 NEWSKTYFDA----------------------------------------- 349
hsTFB2    385 --------------YKWLYDE------------------------------ 391
rnTFB2    384 --------------FKWIYDY------------------------------ 390
dmTFB2    391 --------------ESSFLASLE---------------------------- 399
ptTFB2    382 --------------YKWLYDE------------------------------ 388
mmuTFB2   383 --------------FKWIYDY------------------------------ 389
btTFB2    381 --------------CKWLYDE------------------------------ 387
trTFB2    309 --------------QSWLYEE------------------------------ 315
tnTFB2    461 --------------QSWLYEE------------------------------ 467
scTFB     329 --------------MDFVDMY------------------------------ 335
spTFB     356 --------------HLHL-GT------------------------------ 361
klTFB     327 --------------VDFYD-------------------------------- 331
egTFB     325 --------------YDFYD-------------------------------- 329
caTFB     335 --------------------------------------------------- 334
ddTFB     391 IDNEKEEEEEEINNSLLDSYKYQLKEALEKKEKIDLLKQKKLENKDIIDQD 441
tbTFB     459 --------------SQQAXGSG----------------------------- 466
lmTFB     438 --------------QQQPPQEGD---------------------------- 446

cons      868                                                     918
```

```
ceTFB1   354 SADNTWSLKNFNC-------------------------------------- 366
hsTFB1   336 ------------------------KEEDDAEN------------------- 343
dmTFB1   511 --------------------------------------------------- 510
agTFB1   420 --------------------------------------------------- 419
amTFB1   310 --------------------------------------------------- 309
xlTFB1   336 ------------------------TEEEDDLL------------------- 343
trTFB1   329 --------------------------------------------------- 328
ciTFB1   350 ------------------------NGNAKDVLFETAEKNGNSKIEIPTFEG 376
hsTFB2   392 -------------------------------------TLE----------- 394
rnTFB2   391 -------------------------------------CSD----------- 393
dmTFB2   400 ---NALLKLETANDEPNLEDGVTLPEEDDAE---ADEIIEEESPVPATTPV 444
ptTFB2   389 -------------------------------------TLE----------- 391
mmuTFB2  390 -------------------------------------CPE----------- 392
btTFB2   388 -------------------------------------FME----------- 390
trTFB2   316 -------------------------------------ILE----------- 318
tnTFB2   468 -------------------------------------ILE----------- 470
scTFB    336 -------------------------------------QTE----------- 338
spTFB    362 -------------------------------------IIE----------- 364
klTFB    332 ---------------------------------------E----------- 332
egTFB    330 --------------------------------------------------- 329
caTFB    335 ---------------------------------PFKPSVADLYDFDITF-- 350
ddTFB    442 QLKNQYHQQNNNNKSYNIDD-IDLDDDDDGENIDYDQFLNEHFKK------ 485
tbTFB    467 --------------------------------------------------- 466
lmTFB    447 ---GAEAEAG---------------------------SLN----------- 456

cons     919                                                     969

ceTFB1   367 -------S 367
hsTFB1   344 -----YRL 346
dmTFB1   511 APKNVEVL 518
agTFB1   420 ---VKGAA 424
amTFB1   310 -------- 309
xlTFB1   344 -------Q 344
trTFB1   329 -------K 329
ciTFB1   377 IFETTSLK 384
hsTFB2   395 DR------ 396
rnTFB2   394 DSE----L 397
dmTFB2   445 KRRRKASS 452
ptTFB2   392 DR------ 393
mmuTFB2  393 DME----- 395
btTFB2   391 DAL----S 394
trTFB2   319 NTQREGWV 326
tnTFB2   471 NTQREGWV 478
scTFB    339 HS-----G 341
spTFB    365 D------S 366
klTFB    333 NE-----D 335
egTFB    330 ---PSDDQ 334
caTFB    351 -------S 351
ddTFB    486 -------- 485
tbTFB    467 -------T 467
lmTFB    457 AGSPSGAV 464

cons     970          977
```
