# Supplementary material for: Sequence and structural evolution of the KsgA/Dim1 methyltransferase family
Source: BMC Res Notes. 2008 Oct 29;1:108. doi: 10.1186/1756-0500-1-108 (PMC2614427; doi:10.1186/1756-0500-1-108)
Supplement: Additional file 3 — Sequence alignment of Erm enzymes. Erm enzymes were identified using the Nomenclature Center for MLS Genes, maintained by Dr. Marilyn C. Roberst [28]. One member of each class was chosen, with two exceptions. ErmI was not used because a corresponding sequence could not be found. Erm32 was not used because this enzyme methylates G748 rather than A2058 [29]. The structure based sequence alignment was performed with Expresso [18]. Structures used for the alignment were 1QAM[23] and 1YUB[24]. Organisms represented are Staphylococcus aureus, Enterococcus faecalis, Bacillus subtilis, Bacillus licheniformis, Saccharopolyspora erythraea, Bacteroides fragilis, Lysinibacillus sphaericus, Streptomyces thermotolerans, Streptomyces fradiae, Streptomyces coelicolor, Clostridium perfringens, Aeromicrobium erythreum, Lactobacillus reuteri, Streptomyces lincolnensis, Streptomyces viridochromogenes, Micromonospora griseorubida, Corynebacterium jeikeium, Streptomyces ambofaciens, Streptomyces venezuelae, Staphylococcus sciuri, Bacillus clausii, Bacteroides coprosuis, Micrococcus luteus, Mycobacterium tuberculosis, Mycobacterium smegmatis, Mycobacterium fortuitum, Mycobacterium mageritense, and Mycobacterium abscessus, Accession numbers are found in Additional file 4. [file 1756-0500-1-108-S3.pdf]

```
T-COFFEE, Version_5.31Fri Oct 26 17:01:36 2007
   Cedric Notredame
   CPU TIME:94 sec.
   SCORE=42
   *
    BAD AVG GOOD
   *
   ErmA     :   43
   ErmB     :   39
   ErmC     :   43
   ErmD     :   41
   ErmE     :   43
   ErmF     :   42
   ErmG     :   42
   ErmH     :   44
   ErmN     :   40
   ErmO     :   42
   ErmQ     :   41
   ErmR     :   42
   ErmS     :   43
   ErmT     :   43
   ErmU     :   43
   ErmV     :   42
   ErmW     :   43
   ErmX     :   45
   ErmY     :   42
   ErmZ     :   37
   Erm30    :   44
   Erm31    :   44
   Erm33    :   43
   Erm34    :   41
   Erm35    :   41
   Erm36    :   44
   Erm37    :   36
   Erm38    :   45
   Erm39    :   45
   Erm40    :   43
   Erm41    :   38
   cons     :   42
```

```
ErmA     1  ------------------------------------------------------  0
ErmB     1  ------------------------------------------------------  0
ErmC     1  ------------------------------------------------------  0
ErmD     1  MKKK----------------------NHKYRGK---------------------  11
ErmE     1  MSSSDEQPRP--------------------RRRNQ--DRQ----HPNQNRPVLG  28
ErmF     1  ------------------------------------------------------  0
ErmG     1  ------------------------------------------------------  0
ErmH     1  MAALLKRILRRR-------------MAEKRSGRGRMAAARTTGAQSRKTAQRSG  41
ErmN     1  MPSRPRTDSP----------------------------------HRHEGPAGPA  20
ErmO     1  MA----------------------------------------------------  2
ErmQ     1  MKAK----------------------SNN-------------------------  7
ErmR     1  MAGP----------------------QDRPRGRGP--SSGRPQRPVGG------  24
ErmS     1  MARAPRSPHPARSRETSRAHPPYGTRADRAPGRGR--DRDRSPDSPGNTSSRDG  52
ErmT     1  ------------------------------------------------------  0
ErmU     1  ------------------------------------------------------  0
ErmV     1  MA----------------------------------------------------  2
ErmW     1  MSSI-------------------------R-----------RRHAAAS------  12
ErmX     1  ------------------------------------------------------  0
ErmY     1  ------------------------------------------------------  0
ErmZ     1  MTLK----------------------SPLP-----------PQSVSAP------  15
Erm30    1  MAMRDSIP---------------------------------------------R  9
Erm31    1  -----------------------------------------------------M  1
Erm33    1  ------------------------------------------------------  0
Erm34    1  MTKK----------------------MNKYNGK---------------------  11
Erm35    1  ------------------------------------------------------  0
Erm36    1  ------------------------------------------------------  0
Erm37    1  ------------------------------------------------------  0
Erm38    1  ------------------------------------------------------  0
Erm39    1  ------------------------------------------------------  0
Erm40    1  ------------------------------------------------------  0
Erm41    1  ------------------------------------------------------  0

cons     1                                                          54
```

```
ErmA     1  ----MNQKNPKDTQNFITSKKHVKEILN----HTNI---------SKQDNVIEIG   38
ErmB     1  -----MNKNIKYSQNFLTSEKVLNQIIK----QLNL---------KETDTVYEIG   37
ErmC     1  ----MNEKNIKHSQNFITSKHNIDKIMT----NIRL---------NEHDNIFEIG   38
ErmD    12  KLN-RGESPNFSGQHLMHNKKLIEEIVD----RANI---------SIDDTVLELG   52
ErmE    29  RTE-RDRNRRQFGQNFLRDRKTIARIAE----TAEL---------RPDLPVLEAG   69
ErmF     1  MTK-KKLPVRFTGQHFTIDKVLIKDAIR----QANI---------SNQDTVLDIG   41
ErmG     1  ----MNKVNIKDSQNFITSKYHIEKIMN----CISL---------DEKDNIFEIG   38
ErmH    42  RSE-ADRRRRVHGQNFLVDRETVQRFVR----FADP---------DPGEVVLEVG   82
ErmN    21  RLD-RDEARRVWGQNFFRSAGSARRFARQLTGAESA---------GNDSVTVEVG   65
ErmO     3  --R-PTQRARTLSQNFLADRATAERVAH----LAVP-------DRGRRPLLLEVG   43
ErmQ     8  --Y-RGKVDISVSQNFITSKNTIYKLIK----KTNI---------SKNDFVIEIG   46
ErmR    25  RSQ-RDRDRRVLGQNFLRDPATIRRIAD----AADV---------DPDGLVVEAG   65
ErmS    53  GRS-PDRARRELSQNFLARRAVAERVAR----LVRP-------AP--GGLLLEVG   93
ErmT     1  ----MNKKNIKDSQNFITSKHHINEILR----NVHL---------NTNDNIIEIG   38
ErmU     1  MPS-RYGSRQDLGQNFLVDPDIIKLIRR----APNE---------RKVPSL--IW   39
ErmV     3  --R-PSRVSRALSQNFLADRAAAGQLAR----LAAP-------HGLPVPLLLEVG   43
ErmW    13  LDTPAVGGRHELGQNFLVDRGVCTRIAE----VVSS---------TTAHPVLELG   54
ErmX     1  MST-YGYGRHEHGQNFLTDHKIINSIVD----LVKQ---------TSG-PIIEIG   40
ErmY     1  ----MNKKDIKFSQNFITSKRHINKIMS----NLEL---------NRNDNVFEIG   38
ErmZ    16  ADS-RSTARREWGQNFFRTAAAACRFSA----QLDGSDTIPPDSPNDLMTVEIG   64
Erm30   10  RAD-RDTLRRELGQNFLQDDRAVRNLVT----HVEG---------DGR-NVLEIG   49
Erm31    2  AFS-PQGGRHELGQNFLVDRSVIDEIDG----LVAR---------TKG-PILEIG   41
Erm33    1  ----MNKKNIKDSQNFITSKRNIDKIMT----NISL---------NEHDNIFEIG   38
Erm34   12  KLS-RGEPPNFSGQHFMHNKRLLKEIVD----KADV---------SVRDTVLELG   52
Erm35    1  MTK-KKLPVRFTGQHFTIDKVLIKDAIK----ESNI---------NQHDTVLDIG   41
Erm36    1  MPT-YRGGRHEHGQNFLTDHTTIDRLSR----LVGD---------STG-PIVEIG   40
Erm37    1  MSA-LGRSRRAWGWHRLHDEWAAR-VVS----AAAV---------RPGELVFDIG   40
Erm38    1  MST-PHHGRHELGQNFLSDRRVIADIVE----IVSR---------TNG-PIIEIG   40
Erm39    1  MSS-VHHGRHENGQNFLRDRRVVGDIVR----MVSH---------TAG-PIVEIG   40
Erm40    1  MSS-KNQGRHEHGQNFLCDRRVVADIVK----IVSH---------TTG-SIVEIG   40
Erm41    1  ---------------------------------------------------MDLG    4

cons    55                                                          108
```

```
ErmA    39  SGKGHFTKELVKMSRSVTAIEIDGGLCQVTKEAVN------PSENIKVIQTDIL   86
ErmB    38  TGKGHLTTKLAKISKQVTSIELDSHLFNLSSEKLK------LNTRVTLIHQDIL   85
ErmC    39  SGKGHFTLELVQRCNFVTAIEIDHKLCKTTENKLV------DHDNFQVLNKDIL   86
ErmD    53  AGKGALTTVLSQKAGKVLAVENDSKFVDILTRKTA------QHSNTKIIHQDIM  100
ErmE    70  PGEGLLTRELADRARQVTSYEIDPRLAKSLREKLS------GHPNIEVVNADFL  117
ErmF    42  AGKGFLTVHLLKIANNVVAIENDTALVEHLRKLFS------DARNVQVVGCDFR   89
ErmG    39  AGKGHFTAELVKRCNFVTAIEIDSKLCEVTRNKLL------NYPNYQIVNDDIL   86
ErmH    83  AGNGAITRELARLCRRVVAYEIDRHFADRLREATA------EDPRIEVVAGDFL  130
ErmN    66  PGAGRITKELVRDGHPIVAVEVDPHWADRLAE--L------ELPNLTVVNDDFT  111
ErmO    44  AGNGALTEPLARRSRELHAYEIDPRLVPGLRARFA------RSPHVHVVAGDFL   91
ErmQ    47  PGKGHITEALCEKSYWVTAIELDRSLYGNLINKFK------SKNNVTLINKDFL   94
ErmR    66  PGEGLLTRELARRAGRVRTYELDQRLARRLSTDLA------QETSIEVVHADFL  113
ErmS    94  AGRGVLTEALAPYCGRLVAHEIDPRLLPALRDRFG----GPHHAHVRISGGDFL  143
ErmT    39  SGKGHFSFELAKRCNYVTAIEIDPKLCRITKNKLI------EYENFQVINKDIL   86
ErmU    40  RRRGHVTLPLSRLGRPVTAVELDPRRVKRLSAR---------APENVKVVGEDIL   85
ErmV    44  AGKGALTELLAPRCRSLLAYEIDPRLVPVLRSRFA------DAPHVRVLGEDFL   91
ErmW    55  AGDGAITRALVAANLPVTALELDPRRVRRLQRTFA--------DGVTVVHGDML  100
ErmX    41  PGSGALTHPISHLGRAITAVEVDAKLAAKLTKKT-------ASASVEVVHDDFL   87
ErmY    39  SGKGHFTLELVQKCNYVTVIEIDSNLCIQTQNKVT------NYDNFRIINKDIL   86
ErmZ    65  AGSGRVTKVLASPGTPLLAVEIDPRWARRLAAE--------SLPDVTVVNEDFL  110
Erm30   50  PGKGAITEELVRSFDTVTVVEMDPHWAAHVRRKF-------EGERVTVFQGDFL   96
Erm31   42  PGDGALTLPLSRHGRPITAVELDGRRAQRLGART--------PGHVTVVHHDFL   87
Erm33   39  SGKGHFTLELVQRCNFVTAIEIDHKLCKTTENKLV------DHDNFQVLNKDIL   86
Erm34   53  AGKGALTTILSERADRVLAVEYDQKCIEALQWKLV------GSKNVSILHQDIM  100
Erm35   42  AGKGFLTVHLLKNVDKVIAIENDVALSQHLRKKFI------HAQNVQVVSCDYR   89
Erm36   41  PGQGRLTRELQKLGRSLTAVEIDSRLADRLASASQ----FREQKHVTVVNADFL   90
Erm37   41  AGEGALTAHLVRAGARVVAVELHPRRVGVLRERF---------PGITVVHADAA   85
Erm38   41  AGDGALTIPLQRLARPLTAVEVDARRARRLAQRTARSAPGPASRPTEVVAADFL   94
Erm39   41  AGDGALTLPLQRLGRPLTAIEIDLHRARRLAD----------RTTAEVIATDFL   84
Erm40   41  AGDGALTVPMQRLGRPLTAIEIDRRRAERLARRT----------TAHVVTADFL   84
Erm41    5  AGHGALTAHLVAAGARVLAVELHPGRARHLRSRFA------E-EDVRVAEADLL   51

cons   109     * .:  :      :   * .                        :   *    162
```

```
ErmA     87  KFSFPKHINYKIYGNIPYNISTDIVKRITF--E--SQAKYSYLIVEKGFAKRLQ  136
ErmB     86  QFQFPNKQRYKIVGSIPYHLSTQIIKKVVF--E--SHASDIYLIVEEGFYKRTL  135
ErmC     87  QFKFPKNQSYKIFGNIPYNISTDIIRKIVF--D--SIADEIYLIVEYGFAKRLL  136
ErmD    101  KIHLPK-EKFVVVSNIPYAITTPIMKMLLN--NPASGFQKGIIVMEKGAAKRFT  151
ErmE    118  TAEPPP-EPFAFVGAIPYGITSAIVDWCLE--A--PTIETATMVTQLEFARKRT  166
ErmF     90  NFAVPK-FPFKVVSNIPYGITSDIFKILMF--ESLGNFLGGSIVLQLEPTQKLF  140
ErmG     87  KFTFPSHNPYKIFGSIPYNISTNIIRKIVF--E--SSATISYLIVEYGFAKRLL  136
ErmH    131  KTSQPK-VPFSVVGNIPFGNTADIVDWCLN--A--RRLRTTTLVTQLEYARKRT  179
ErmN    112  TWPLPD-GPLRFIGNLPFGTGTRMLRRCLALGP--DRCREGVFLLQKQYTRKRT  162
ErmO     92  TARPPR-TPFAVAGNVPFSRTADIVDWCLT--A--PGLTDATLLTQLEYARKRT  140
ErmQ     95  NWKLPKKREYKVFSNIPFYITTKIIKKLLL--EELNSPTDMWLVMEKGSAKRFM  146
ErmR    114  TAPHPE-EPFQFVGAIPYGITSAIVDWCLT--A--PTLTSATLVTQQEFARKRT  162
ErmS    144  AAPVPR-EPFALAGNIPYSRTAGIVDWALR--A--RTLTSATFVTQLEYARKRT  192
ErmT     87  QFKFPKNKSYKIFGNIPYNISTDIIRKIVF--E--STATESYLIVEYGFAKRLL  136
ErmU     86  RFRLPT-VPHTVVGNIPFHVTTATMRRILV--A--PAWVSAVLVVQWEVARRRA  134
ErmV     92  RARAPR-TPFSVAGNVPFSRTAAVVAWCLR--A--PHLTDATLLTQLEYARRRT  140
ErmW    101  RYDFGP-YPHHVVSTVPFSITTPLLRRLIG--Q--RFWHTAVLLVQWEVARKRA  149
ErmX     88  NFPLPA-TPCVIVGNIPFHLTTAILRKLLH--A--PAWTDAVLLMQWEVARRRA  136
ErmY     87  QFKFPNNKAYKIYGNIPYYISTDIVRKIVF--E--SEATVSYLIVEEGFAKRLL  136
ErmZ    111  TLQLPG-QPVRLIGNLPFVTGTRMLRRCLD--MGPARMRQGVFLLQREYVGKRT  161
Erm30    97  DFRIPR-DIDTVVGNVPFGITTQILRSLLE--S--TNWQSAALIVQWEVARKRA  145
Erm31    88  QYPLPR-NPHVVVGNVPFHLTTAIMRRLLD--A--QHWHTAVLLVQWEVARRRA  136
Erm33    87  QFKFPKNQSYNIFGNIPYNISTDIVKRITF--E--SQAKYSYLIVEKGFAKRLQ  136
Erm34   101  KVALPT-EPFVVVSNIPYSITTAIMKMLLN--NPKNKLQRGAIVMEKGAAKRFT  151
Erm35    90  NFVVPK-VPFKVVSNIPFGITSDIFSSLMF--ENVEYFLCGSIILQSEPAKKLF  140
Erm36    91  HWPLPT-TPYVVVGNVPFHLTTAILRRLLH--D--GAWTQVVLLVQWEVARRRA  139
Erm37    86  SIRLPG-RPFRVVANPPYGISSRLLRTLLA--PNS-GLVAADLVLQRALVCKFA  135
Erm38    95  RYPLPR-SPHVVVGNLPFHLTTAILRRLLH--G--PGWTTAVLLMQWEVARRRA  143
Erm39    85  RYRLPR-TPHVVVGNLPFHLTTAILRRLLH--E--NGWTDAILLVQWEVARRRA  133
Erm40    85  RYRLPP-TEHVVVGNLPFHLTTAILRRLLH--S--PAWTDAVLLMQWEVARRRA  133
Erm41    52  AFRWPR-RPFRVVASPPYQVTSALIRSLLT--P-ESRLLAADLVLQRGAVHKHA  101

cons    163             . .  *:   :  .                 :: :     :    216
```

```
ErmA   137 N-LQR--ALGLLLMVEMDIKMLKKVPPLYFHPKPSVDSVLIVLERH-QPLI-SK 185
ErmB   136 D-IHR--TLGLLLHTQVSIQQLLKLPAECFHPKPKVNSVLIKLTRH-TTDV-PD 184
ErmC   137 N-TKR--SLALFLMAEVDISILSMVPREYFHPKPKVNSSLIRLNRK-KSRI-SH 185
ErmD   152 SK-FIKNSYVLAWRMWFDIGIVREISKEHFSPPPKVDSAMVRITRKKDAPLS-H 203
ErmE   167 GDYGRWSRLTVMTWPLFEWEFVE-----------KVDSAIMRLRRRAEPLLE-G 208
ErmF   141 SR-KLYNPYTVFYHTFFDLKLVYEVGPESFLPPPTVKSALLNIKRKH-LFF-DF 191
ErmG   137 D-TNR--SLALLLMAEVDISILAKIPRYYFHPKPKVDSALIVLKRK-PAKM-AF 185
ErmH   180 GGYRRWSRLTVATWPEVEWRMGERISRRWFRPVPAVDSAVLRLERRPVPLIP-P 232
ErmN   163 GAYG-GNLFNAQWEPWYTFRRGLGFPRQEFAPVPGSDTETLLVRSRPRPLAP-W 214
ErmO   141 GDYGRWTLLTVLTWPRHEWRLVGRVGRSRFCPAPRVDAGILRIERRPTALLTGA 194
ErmQ   147 G-IPRESKLSLLLKTKFDIKIVHYFNREDFHPMPSVDCVLVYFKRKYKYDI-SK 198
ErmR   163 GDYGRWTALTVTTWPTFEWQYVAKVDRTLFTPVPRVHSAIMRLRRRPQPLLRDA 216
ErmS   193 GDYGRWSLLTVRTWPRHEWRLLGRVSRREFRPVPRVDSGILRIERRERPLLP-S 245
ErmT   137 N-TNR--SLALFLMTEVDISILSKIPREYFHPKPRVNSSLIVLKRH-PSKI-SL 185
ErmU   135 G-IGGCSLVTAESWPWFDFSVLKRVPRFAFRPAPSVDGGILVIERRPEPLV--- 184
ErmV   141 GDYGSWTRLTVLTWPRHEWRLAGRVGRRSFRPVPRVDAGIVRIERRRTPLLA-P 193
ErmW   150 G-VGGTTMLTAASWPWYEFTLVERVPKTSFDPVPSVDGGILVIERRSAPLL-DD 201
ErmX   137 G-VGASTMMTAQWSPWFTFHLGSRXPRSAFRPQPNVDGGILVIRRVGDPKIP-I 188
ErmY   137 N-TNR--SLALLLMTEVDISILSKIPKEYFHPKPKINSSLIILKRH-PSKI-SY 185
ErmZ   162 G-AWGGNLFNAQWEPWYSFDRGLAFSRQDFTPVPRADTQTLMVAPHRRPSV-PW 213
Erm30  146 G-RSGGSLLTTSWAPWYEFAVHDRVRASSFRPMPRVDGGVLTIRRRPQPLLP-E 197
Erm31  137 G-VGGSTLLTAGWAPWYEFDLHSRVPARAFRPMPGVDGGVLAIRRRSAPLV--- 186
Erm33  137 N-LQR--ALGLLLMVEMDIKMLKKVPPLYFHPKPSVDSVLIVLERH-QPLI-SK 185
Erm34  152 S-VSPKDAYVMAWHMWFDIHYERGISRSSFSPPPKVDSALVRIVRKQHPLFP-Y 203
Erm35  141 SS-KVYNPLTVLYHTYYDLKFLYEINPESFLPPPTVKSALLRIERKQ-ISL-DI 191
Erm36  140 G-IGGSSMMTAQWWPWIDFSLHGRVPRSAFKPAPSVDGGLLEMTRRPDPLLS-P 191
Erm37  136 SRNA-----------RRFTLTVGLMLPRRAFLPPPHVDSAVLVVRRRKC------ 173
Erm38  144 A-VGGATMMTAQWWPWFEFGLARKVSAASFTPRPAVDAGLLTITRRSRPLVD-V 195
Erm39  134 G-VGGATMMTAQWWPWFEFGLARKVSADAFRPRPSVDAGLLTIQRRAEPLLP-W 185
Erm40  134 A-VGGATMMTAQWWPWFEFGLARKVSADAFRPRPSVDAGLLTITRRREPLID-G 185
Erm41  102 ------KRAPVRHW---TLRAGITLPRSAFHHPPQVDSSVLVIRRR-------- 138

cons   217                                     .   : .            270
```

```
ErmA    186  KDYKKYRSFVYKWVN-------REYRVLF---TKNQFRQALKHANV---TNINK  226
ErmB    185  KYWKLYTYFVSKWVN-------REYRQLF---TKNQFHQAMKHAKV---NNLST  225
ErmC    186  KDKQKYNYFVMKWVN-------KEYKKIF---TKNQFNNSLKHAGI---DDLNN  226
ErmD    204  KHYIAFRGLAEYALKEPNIPLCVALRGIF---TPRQMKHLRKSLKINNEKTVGT  254
ErmE    209  AALERYESMVELCFTGVGGNIQASLLRKY---PRRRVEAAFDHAGVGGGAVVAY  259
ErmF    192  KFKAKYLAFISYLLEKPDLSVKTALKSIF---RKSQVRSISEKFGLNLNAQIVC  242
ErmG    186  KERKKYETFVMKWVN-------KEYEKLF---TKNQFNKALKHARI---YDINN  226
ErmH    233  GLMHDFRDLVETGFTGKGGSLDASLRRRF---PARRVAAGFRRARLEQGVVVAY  283
ErmN    215  SRHAAYQRFVEDVFNTSRLTIGEAAR-AL---DRRAGPGWLRGARVPPGLRVKD  264
ErmO    195  AARRDWADLVELGFSGVGGSLHASLRRAH---SRRRVDAAFRAARLDPGVLVGE  245
ErmQ    199  DEWNEYTSFISKSIN--------NLRDVF---TKNQIHAVIKYLGINL-NNISE  240
ErmR    217  AARSRFADMVEIGFVGKGGSLYRSLTREW---PRSKVDSAFARADVHHDEIVAF  267
ErmS    246  AALGDYHRMVELGFSGVGGSLYASLRRAH---RAGPLDAAFRAARLDRSVVVAY  296
ErmT    186  KDRKQYENFVMKWVN-------KEYIKLF---SKNQFYQALKYARI---DDLNN  226
ErmU    185  RERREYQDFVRQVFTGRGHGLREILQRIG-RVQDSDLSAWFRAHGVSPQALPKD  237
ErmV    194  GADAGWRELVDLGFSGAGGSLHASLRRAR---PRRRVDAAFRAAGLDRDVLVGE  244
ErmW    202  RCVGDYQNLVREVYTGPGRGLAAILRTRL---PGREVDAWLRRERVDPAALPRD  252
ErmX    189  EQRKAFQAMVHTVFTARGRGIGEILRRAGLFSSRSETQSWLRSRGIDPATLPPR  242
ErmY    186  KDKKMYNNFVMKWVN-------QEYSKLF---TKNQFNKALNYAKI---KDLKN  226
ErmZ    214  REKAAYQRFVQRVFDTGQMTVGDAARKVL---RRG-HAQFVRGAGVRPADRVKD  263
Erm30   198  SASRAFQNFAEAVFTGPGRGLAEILRRH---IPKRTYRSLADRHGIPDGGLPKD  248
Erm31   187  GQVKTYQDFVRQVFTGKGNGLKEILRRTG-RISQRDLATWLRRNEISPHALPKD  239
Erm33   186  KDYKKYRSFVYKWVN-------REYRVLF---TKNQFRQALKHANV---TNINK  226
Erm34   204  KEAKAMHDFLSYALNNPRAPLDQVLRGIF---TAPQAKKVRQAIGVKPETPVAM  254
Erm35   192  GLKVKYLNFVSYMLQKPDLTVKTAMKSIF---RKKQVRSISEKFGVDLNSKIVC  242
Erm36   192  DARESYRQFVHDVFTSRGRGIGEILANVSSSLGKRGALQLLKSEGIRSSSLPKD  245
Erm37   174  ------------------------------------------------------  173
Erm38   196  ADRARYQALVHRVFTGRGHGMAQILQRL----PTPVPRTWLRANGIAPNSLPRQ  245
Erm39   186  ADRRAYQALVHRVFTGRGRGLAQILR-------PHVHPRWLSANGIHPSALPRA  232
Erm40   186  ADRRRYQALVHAVFTGRGRGVAQIVGPR-------VPRHWLRHNGITPSALPRD  232
Erm41   139  ------------------------------------------------------  138

cons    271                                                        324
```

```
ErmA    227  LSKEQFLSIFNSYKLFH-------------------------------------  243
ErmB    226  VTYEQVLSIFNSYLLFN-G-----------------------------------  243
ErmC    227  ISFEQFLSLFNSYKLFN-K-----------------------------------  244
ErmD    255  LTENQWAVIFNTMTQYVMH-----------------------------------  273
ErmE    260  VRPEQWLRLFERLDQKNEPRGGQPQRGRRTGGRDHGDRRTGGQDRGDRRTGGRD  313
ErmF    243  LSPSQWLNCFLEMLEVVPEKF--------------H------------------  264
ErmG    227  ISFEQFVSLFNSYKIFN-G-----------------------------------  244
ErmH    284  VTPGQWITLFEELH----------------------------------------  297
ErmN    265  ITAEQWADLFHACTPPPA-RRISPQRRR--------------------------  291
ErmO    246  VAPDRWLRLHEELTA---------------------------------------  260
ErmQ    241  VSYNDWIQLFRYKQKI--D-----------------------------------  257
ErmR    268  VHPDQWITLFQLLDGSRGGAA-----------------RGPGDQRGR------R  298
ErmS    297  VTPEQWLTVFRTLRPVRS------------------------------------  314
ErmT    227  ISFEQFLSLFNSYKLFN-R-----------------------------------  244
ErmU    238  LTAEQWASLWGMARGGR----------------------SVPRTRRP-------  262
ErmV    245  VPPWTWLRLHEVLG----------------------------------------  258
ErmW    253  LKAGHWASLYRLYREVGTR--------------------PAPAGRSV------R  280
ErmX    243  LHTSDWIDLFQVTGSSPP------------------------------------  260
ErmY    227  INFEQFLSVFNSYKLFN-N-----------------------------------  244
ErmZ    264  LTVPEWTALFRAYGRTADR-----------------------------------  282
Erm30   249  LTLTQWIALFQASQPSYAPGAPG--------------TRMPGQGGG------AG  282
Erm31   240  LKPGQWASLWELTGGTADGSFDGTA----------GGGAAGS--HGAARVG--A  279
Erm33   227  LSKEQFLSIFNSYKLFH-------------------------------------  243
Erm34   255  LHARQWAMVCDAMVRHVPKVY--------------W------------------  276
Erm35   243  LTPNQWKNCFLEMLEVVPEKF--------------H------------------  264
Erm36   246  LSAEQWARLFTSASPTKSAKT--------------GRNAHPA--HSARRQGR--  281
Erm37   174  ---GDWQG----------------------------------------------  178
Erm38   246  LSAAQWAALFEQTRLTGAQRV--------------DRPRDVQHGRAHRRRGGEV  285
Erm39   233  LTARQWVALFDAAG----------------------------------------  246
Erm40   233  LTAAQWAALFEVTSEAKR------------------------------------  250
Erm41   139  ------------------------------------------------------  138

cons    325                                                        378
```

```
ErmA    244  ------------------------------------------------------  243
ErmB    244  ------------------------------------------------------  243
ErmC    245  ------------------------------------------------------  244
ErmD    274  -----------------------------------HKW-------PRANKRKP-  284
ErmE    314  HRDRQASGHGDRRSSGRNRDDGRTGEREQGDQGGRR------------------  349
ErmF    265  ------------------------------------------------------  264
ErmG    245  ------------------------------------------------------  244
ErmH    298  ------------------------------------------------------  297
ErmN    292  ------------------------------------------------------  291
ErmO    261  ------------------------------------------------------  260
ErmQ    258  ------------------------------------------------------  257
ErmR    299  GRPGGGPRPDGRAGGGPRRDAG--GRRTGDGRGGRPRP-------PRG----G-  338
ErmS    315  -RPA--------------------------------------------------  317
ErmT    245  ------------------------------------------------------  244
ErmU    263  --------------------------RGLPPRT------------SRGPRRNS-  277
ErmV    259  ------------------------------------------------------  258
ErmW    281  ARPG---------------SVG--PDRSLPPRGLRSGP-------PRARRRGG-  309
ErmX    261  --------------------RH--RPISQSGSSQR-----PPQRKNRGRRR---  284
ErmY    245  ------------------------------------------------------  244
ErmZ    283  ------------------------------------------------------  282
Erm30   283  GRDYDSETSRAAVPGS---------RRYGPTRGGEPCAPRAQVRQTKGRQ----  323
Erm31   280  GHPGGRVS------------------------ASRRGV--PQARRGR-------  300
Erm33   244  ------------------------------------------------------  243
Erm34   277  ---------------------------------------------PRRKR----  281
Erm35   265  ------------------------------------------------------  264
Erm36   282  ------------------------------------------------------  281
Erm37   179  ------------------------------------------------------  178
Erm38   286  DRPATHHKQTGPVVGQRQPQRG--RDADADPDDQRTAP--PVTRHHQGERRDED  335
Erm39   247  ------------------------------------------------------  246
Erm40   251  --------------------------------------------------C---  251
Erm41   139  ------------------------------------------------------  138

cons    379                                                         432
```

```
ErmA   244 ---------------------------------------------------  243
ErmB   244 -------------------------------------------------RK  245
ErmC   245 ---------------------------------------------------  244
ErmD   285 ------------------------------------------------GEI  287
ErmE   350 ------GPSGGGRTGGRPGRRGGP------------------------GQR  370
ErmF   265 -------------------------------------------------PS  266
ErmG   245 ---------------------------------------------------  244
ErmH   298 ------------------------------------------------G-R  299
ErmN   292 ---------------------------------------------------  291
ErmO   261 ---------------------------------------------------  260
ErmQ   258 ---------------------------------------------------  257
ErmR   339 ------------------------------------------------Q-A  340
ErmS   318 ------------------------------------------------G-R  319
ErmT   245 ---------------------------------------------------  244
ErmU   278 ------------------------------------------------G--  278
ErmV   259 --------------------------------------------------S  259
ErmW   310 ------------------------------------------------G-A  311
ErmX   285 ---------------------------------------------------  284
ErmY   245 ---------------------------------------------------  244
ErmZ   283 ---------------------------------------------------  282
Erm30  324 ---GARGSSYGRRTG-----------------------------------R  336
Erm31  301 --GHAVRSSTGT----EPRWGRGRAESA-----------------------  322
Erm33  244 ---------------------------------------------------  243
Erm34  282 ---------------------------------------------------  281
Erm35  265 -------------------------------------------------PS  266
Erm36  282 ---------------------------------------------------  281
Erm37  179 --------------------------------------------------R  179
Erm38  336 QADHQDRPLTGEHLAGEFLWRHASFDSSASTTLVSRKARVNGPTPPGLGDT  386
Erm39  247 ---------------------------------------------------  246
Erm40  252 ---------------------------------------------------  251
Erm41  139 ---------------------------------------------------  138

cons   433                                                      483
```
