# Supplementary material for: Sequence and structural evolution of the KsgA/Dim1 methyltransferase family
Source: BMC Res Notes. 2008 Oct 29;1:108. doi: 10.1186/1756-0500-1-108 (PMC2614427; doi:10.1186/1756-0500-1-108)
Supplement: Additional file 4 — Sequences used in protein alignments. Sequences were compiled from NCBI and Ensembl; organisms and accession numbers are indicated. [file 1756-0500-1-108-S4.pdf]

Organism	Domain	KsgA/Dim1	mtTFB	mtTFB1	mtTFB2	Pfc1
Arabidopsis thaliana (at)	Eukarya	NP_182264				NP_171690
Dictyostelium discoideum (dd)	Eukarya	XP_638864	XP_637775			
Leishmania brazilensis (lb)	Eukarya	XP_001566655				
Giardia lamblia (gl)	Eukarya	XP_001704233				
Plasmodium vivax (pv)	Eukarya	XP_001616795				
Homo sapiens (hs)	Eukarya	NP_055288		NP_057104	NP_071761	
Saccharomyces cerevisiae (sc)	Eukarya	NP_015057	P14908			
Drosophila melanogaster (dm)	Eukarya			NP_996062	NP_649971	
Caenorhabditis elegans (ce)	Eukarya			NP_491242		
Methanopyrus kandleri (mk)	Archaea	P_614217				
Methanosaeta thermophila (mth)	Archaea	YP_843421				
Haloquadratum walsbyi (hw)	Archaea	YP_658586				
Methanoculleus marisnigri (mma)	Archaea	YP_001047866				
Methanocaldococcus jannaschii (mj)	Archaea	NP_248023				
Pyrococcus horikoshii (ph)	Archaea	NP_143656				
Methanosphaera stadtmanae (ms)	Archaea	YP_448554				
Picrophilus torridus (pt)	Archaea	YP_023164				
Archaeoglobus fulgidus (af)	Archaea	NP_070611				
Aeropyrum pernix (ap)	Archaea	NP_147322				
Sulfolobus solfataricus (ss)	Archaea	NP_342254				
Pyrobaculum aerophilum (pa)	Archaea	NP_560585				
Cenarchaeum symbiosum (cs)	Archaea	YP_875407				
Synechococcus elongatus (se)	Bacteria	YP_171912				
Bacillus subtilis (bs)	Bacteria	NP_387923				
Mycobacterium tuberculosis (mtu)	Bacteria	NP_215526				
Thermus thermophilus (tt)	Bacteria	YP_005890				
Bacteroides fragilis (bf)	Bacteria	YP_098063				
Chlamydia trachomatis (ct)	Bacteria	NP_219862				
Borrelia burgdorferi (bb)	Bacteria	NP_212724				

Escherichia coli (ec)	Bacteria	NP_414593				
Anopheles gambiae (ag)	Eukarya			XP_320403		
Apis mellifera (am)	Eukarya			XP_001121333		
Xenopus laevis (xl)	Eukarya			NP_001079850		
Takifugu rubripes (tr)	Eukarya			ENSTRUP00000024475*	ENSTRUP00000026212*	
Ciona intestinalis (ci)	Eukarya			ENSCINP00000008063*		
Rattus norvegicus (rn)	Eukarya				P_001008294	
Pan troglodytes (pt)	Eukarya				XP_514318	
Mus musculus (mmu)	Eukarya				NP_032275	
Bos taurus (bt)	Eukarya				NP_001033216	
Tetraodon nigroviridis (tn)	Eukarya				CAG11026	
Schizosaccharomyces pombe (sp)	Eukarya		NP_593495			
Kluyveromyces lactis (kl)	Eukarya		XP_451899			
Eremothecium gossypii (eg)	Eukarya		NP_986111			
Candida albicans (ca)	Eukarya		XP_722572			
Trypanosoma brucei (tb)	Eukarya		AAM97535			
Leishmania major (lm)	Eukarya		XP_843186			
Erm enzymes						
Organism	Protein	Accession				
Staphylococcus aureus	ErmA	CAA26964				
Enterococcus faecalis	ErmB	CAA68299				
Bacillus subtilis	ErmC	P13956				
Bacillus licheniformis	ErmD	AAA22599				
Saccharopolyspora erythraea	ErmE	AAA26492				
Bacteroides fragilis	ErmF	AAA98217				
Lysinibacillus sphaericus	ErmG	AAA22419				
Streptomyces thermotolerans	ErmH	AAC32026				

Streptomyces fradiae	ErmN	CAA66307				
Streptomyces coelicolor	ErmO	NP_630197				
Clostridium perfringens	ErmQ	AAC36915				
Aeromicrobium erythreum	ErmR	P09891				
Streptomyces fradiae	ErmS	AAA26742				
Lactobacillus reuteri	ErmT	Q00014				
Streptomyces lincolnensis	ErmU	AAB23456				
Streptomyces viridochromogenes	ErmV	AAB51440				
Micromonospora griseorubida	ErmW	BAA03402				
Corynebacterium jeikeium	ErmX	AAK28910				
Staphylococcus aureus	ErmY	BAB20748				
Streptomyces ambofaciens	ErmZ	CAM96571				
Streptomyces venezuelae	Erm30	AAC69328				
Streptomyces venezuelae	Erm31	AAC69327				
Staphylococcus sciuri	Erm33	CAC86410				
Bacillus clausii	Erm34	AAP74657				
Bacteroides coprosuis	Erm35	AAK07612				
Micrococcus luteus	Erm36	AAL68827				
Mycobacterium tuberculosis	Erm37	CAA98396				
Mycobacterium smegmatis	Erm38	AAN86837				
Mycobacterium fortuitum	Erm39	AAR92235				
Mycobacterium mageritense	Erm40	AAS76623				
Mycobacterium abscessus	Erm41	ABW06859				

* Indicates Ensembl peptide ID numbers; all others are NCBI accession numbers.
